# EANM consensus document on the use of [^18^F]FDG PET/CT in fever and inflammation of unknown origin

**DOI:** 10.1007/s00259-024-06732-8

**Published:** 2024-04-27

**Authors:** Søren Hess, Edel Noriega-Álvarez, Lucia Leccisotti, Giorgio Treglia, Domenico Albano, Anne Roivainen, Andor W.J.M. Glaudemans, Olivier Gheysens

**Affiliations:** 1 Department of Nuclear Medicine, Odense University Hospital, Odense, Denmark; 2 Department of Clinical Research, Faculty of Health Sciences, University of Southern Denmark, Odense, Denmark; 3 Department of Nuclear Medicine, University Hospital of Guadalajara, Guadalajara, Spain; 4 Section of Nuclear Medicine, Department of Radiological Sciences and Haematology, Università Cattolica del Sacro Cuore, Rome, Italy; 5 Unit of Nuclear Medicine, Fondazione Policlinico Universitario A. Gemelli IRCCS, Rome, Italy; 6 Division of Nuclear Medicine, Imaging Institute of Southern Switzerland, Ente Ospedaliero Cantonale, Bellinzona, Switzerland; 7 Faculty of Biomedical Sciences, Università della Svizzera italiana, Lugano, Switzerland; 8 Faculty of Biology and Medicine, University of Lausanne, Lausanne, Switzerland; 9 Nuclear Medicine, University of Brescia, ASST Spedali Civili Brescia, Brescia, Italy; 10 Turku PET Centre, University of Turku, Turku, Finland; 11 Turku PET Centre, Turku University Hospital, Turku, Finland; 12 InFLAMES Research Flagship, University of Turku, Turku, Finland; 13 Department of Nuclear Medicine and Molecular Imaging, University of Groningen, University Medical Center Groningen, Hanzeplein 1, Groningen, 9700 RB The Netherlands; 14 Department of Nuclear Medicine, Cliniques Universitaires Saint-Luc and Institute of Clinical and Experimental Research (IREC), Université Catholique de Louvain, Brussels, Belgium

**Keywords:** Infection, Inflammation, FDG, PET/CT, FUO, IUO, Fever of unknown origin, Inflammation of unknown origin

## Abstract

**Purpose:**

Patients with fever and inflammation of unknown origin (FUO/IUO) are clinically challenging due to variable clinical presentations with nonspecific symptoms and many differential diagnoses. Positron emission tomography/computed tomography (PET/CT) with 2-deoxy-2-[^18^F]fluoro-D-glucose ([^18^F]FDG) is increasingly used in FUO and IUO, but the optimal diagnostic strategy remains controversial. This consensus document aims to assist clinicians and nuclear medicine specialists in the appropriate use of [^18^F]FDG-PET/CT in FUO and IUO based on current evidence.

**Methods:**

A working group created by the EANM infection and inflammation committee performed a systematic literature search based on PICOs with “patients with FUO/IUO” as population, “[^18^F]FDG-PET/CT” as intervention, and several outcomes including pre-scan characteristics, scan protocol, diagnostic yield, impact on management, prognosis, and cost-effectiveness.

**Results:**

We included 68 articles published from 2001 to 2023: 9 systematic reviews, 49 original papers on general adult populations, and 10 original papers on specific populations. All papers were analysed and included in the evidence-based recommendations.

**Conclusion:**

FUO and IUO remains a clinical challenge and [^18^F]FDG PET/CT has a definite role in the diagnostic pathway with an overall diagnostic yield or helpfulness in 50–60% of patients. A positive scan is often contributory by directly guiding treatment or subsequent diagnostic procedure. However, a negative scan may be equally important by excluding focal disease and predicting a favorable prognosis. Similar results are obtained in specific populations such as ICU-patients, children and HIV-patients.

**Supplementary Information:**

The online version contains supplementary material available at 10.1007/s00259-024-06732-8.

## Introduction

Imaging of infection and inflammation has been part of nuclear medicine since the 1970s with the use of [^67^Ga]Ga-citrate, radiolabelled white blood cells, and finally the glucose analog 2-deoxy-2-[^18^F]-fluoro-*D*-glucose ([^18^F]FDG). In the first decade of [^18^F]FDG PET, incidental [^18^F]FDG uptake in infectious or inflammatory foci in cancer patients was considered a false-positive nuisance, but from the 1990s onward, this changed with a greater understanding and appreciation of [^18^F]FDG PET in infectious and inflammatory conditions [1, 2].

An early application of [^18^F]FDG PET in infectious and inflammatory diseases was fever of unknown origin (FUO), a heterogeneous and clinically challenging condition with a multitude of underlying differential diagnoses, i.e. infections, non-infectious inflammatory diseases (NIID), malignancies, and miscellaneous [3–9]. The definition of FUO has changed several times since the original definition from 1961 [10]; the current definition includes fever > 38.3° C on more than three occasions during more than three weeks with no clear diagnosis despite three days of relevant inpatient workup or three outpatient visits [11].

In recent years, inflammation of unknown origin (IUO) was introduced as an equivalent to FUO without fever, i.e. patients with normal temperature, but increased C-reactive protein (CRP) and/or erythrocyte sedimentation rate (ESR) on more than three occasions for more than three weeks and no diagnosis despite relevant inpatient or outpatient workup similar to FUO.

The overarching etiologies of FUO and IUO (i.e. infection, NIID, malignancies, and miscellaneous) are comparable [12–16], although infections are usually more prevalent in FUO and NIID more prevalent in IUO [17–20]. Therefore, the diagnostic approach is similar in both conditions [21]. It is important to remember that not all patients with unexplained infection or nonspecific systemic inflammation meet the stringent criteria of FUO or IUO, but they may still be suitable candidates for a [^18^F]FDG PET/CT exam.

Identification and accurate localization of the causes of FUO/IUO is essential to initiate appropriate treatment or to guide further diagnostic procedures [22]. Conventional radiology may be of limited use since infectious and inflammatory conditions often cause limited morphologic changes especially at early stages [12, 22]. [^18^F]FDG PET/CT has become the imaging modality of choice in the work-up of patients with FUO/IUO and has replaced scintigraphy with [^67^Ga]Ga-citrate or radiolabeled white blood cells if access to PET/CT is available [23]. The advantages of [^18^F]FDG PET/CT include the possibility to perform a whole-body examination in a single imaging session with good resolution and high sensitivity for detecting low-grade and early stage infection/inflammation with relatively lower radiation exposure compared to a diagnostic contrast-enhanced whole-body CT scan [5, 24, 25]. [^18^F]FDG PET/CT is superior to CT of the chest-abdomen-pelvis in reaching a final diagnosis in FUO; one study reported a diagnostic yield beyond conventional CT estimated at 32% [5], and one showed that [^18^F]FDG PET/CT performed three to four-fold better than CT with regards to diagnostic yield and clinical helpfulness [18]. The better performance has been explained by [^18^F]FDG uptake in the vascular regions [4] or inflammation of the musculoskeletal system [5] that are not easily detected by CT. One study suggests [^18^F]FDG PET/CT as first-line modality especially in FUO-patients with suspected endovascular infection, large vessel vasculitis, and hematological malignancy [26]. The nonspecificity of [^18^F]FDG uptake may be considered an advantage owing to the broad spectrum of differential diagnosis in FUO/IUO. On the other hand, the general limitations of false-positive [^18^F]FDG uptake, false-negative scans, physiologic uptake etc. also apply to infection and inflammation, which may lead to unnecessary invasive investigations or therapy.

The literature on [^18^F]FDG PET in FUO/IUO reflects the heterogeneous nature of the clinical entity itself and comparison across different studies is difficult. It may be difficult to dichotomize findings to obtain sensitivity and specificity, and instead, most studies report either “diagnostic yield” or the proportion of patients in whom FDG-PET/CT was considered “helpful”. However, both terms are defined differently; some consider only true-positive findings helpful, whereas some also include true-negative findings that may rule out focal infection, inflammation, or malignancies with a high negative predictive value. Some define the term “diagnostic yield” as the fraction of true positives among all positive and negative findings, whereas some the term interchangeably with diagnostic helpfulness [23, 27, 28].

. The eldest studies performed with stand-alone PET are now obsolete and not comparable to those performed with modern PET/CT scanners. Most studies are retrospective, with a significant heterogeneity with regard to the use of [^18^F]FDG PET/CT during the workup strategy, and baseline characteristics such as population size, definition of FUO/IUO, inclusion and exclusion criteria, patient demographics, and imaging parameters.

The purpose of this consensus document is to help the clinicians and nuclear medicine specialists involved in the management of patients with FUO/IUO to decide how and when to perform [^18^F]FDG PET/CT in this setting according to the current evidence.

## Methodology (Systematic search of the literature)

To obtain an evidence-based consensus document, the EANM Inflammation & Infection Committee created a working group to perform a systematic search of the literature. The literature search was based on the PICO (Population or problem–Intervention–Comparator–Outcome) strategy. The population has been defined as “patients with FUO/IUO”, the intervention as “[^18^F]FDG PET/CT” and several outcomes were considered including scan protocol, diagnostic yield, impact on management, prognosis, and cost-effectiveness.

A systematic literature search was prepared using two bibliographic databases (PubMed/Medline and Cochrane library) from 2001 up to December 2023. A broad search string using a combination of key words related to elements of the PICO question and in particular the Problem (“fever” OR “pyrexia” OR “FUO” OR “IUO” OR “PUO”) and the Intervention (“FDG” OR “fluorodeoxyglucose” OR “PET”) was used. Cochrane library yielded no results; in PubMed the first retrieved report on PET/CT was from 2006. The complete PubMed search string is presented in Supplemental File 1. We extracted systematic reviews and meta-analyses on the topic of interest as we considered those to be the most relevant evidence-based documents. Furthermore, we extracted all relevant original papers on the subject. Finally, we manually perused the reference lists of all identified admissible papers to identify any further papers. The flowchart for paper selection is presented in Supplemental File 2. All systematic reviews/meta-analyses and original articles (excluding case reports) related to the role of [^18^F]FDG PET/CT in patients with FUO/IUO are listed in a Supplemental file 3 and form the basis of the information reported in this evidence-based document.

## Pre-scan investigations

There is no established consensus on which investigations should be performed prior to [^18^F]FDG PET/CT scan, but the diagnostic workup of FUO/IUO usually begins with routine laboratory tests (including a complete blood count with differential, ESR, and CRP levels), whereas first-line imaging consists of chest X-ray and abdominal ultrasound [29]. More advanced invasive procedures or 3D-imaging modalities such as CT, MRI and/or [^18^F]FDG PET/CT may be guided by symptoms, clinico-biochemical findings, or the physicians preference and are usually considered second-line, with PET/CT usually being performed when first-line work-up is negative or when a final diagnosis is not established. The main recommendations from the literature regarding relevant pre-PET-scan investigations in FUO [7, 16, 19, 29–43] or IUO [14, 16, 19, 32, 33, 44–46] are summarized below.

### Pre-scan laboratory tests

CRP is a well-established nonspecific marker of infection, inflammation, and malignancy with an essential role in clinical routine. CRP is elevated in most cases of prolonged fever, FUO and IUO, but the association between CRP thresholds and contribution of [^18^F]FDG PET/CT remains controversial. Several reports showed a significant association between CRP levels and prediction of usefulness [19, 29, 38, 44, 46–51], while others did not observe this relation [14, 34, 35, 37, 43, 52–54], and similar opposing results were encountered in pediatric patients [33, 41]. The majority of patients in the abovementioned reports had elevated CRP, but no cut-off value has been established to select patients for [^18^F]FDG PET/CT or to predict helpfulness. Some suggest the number of PET/CT positive patients increase with increasing CRP [14, 29], and some studies report that PET/CT-positive findings are very rare in patients with normal CRP-levels [45, 55], but there are also patients in some studies that are PET/CT positive despite normal CRP levels [34, 47].

Other pre-scan laboratory tests showed similar contradictory results: ESR [7, 31, 33, 35, 37, 44], leukocyte count [7, 33, 34, 41], platelets [5, 31], serum procalcitonin [11, 36], and hemoglobin [29, 31, 43]. One study suggested a positive association between the number of inflammatory markers, final diagnosis, and [^18^F]FDG PET/CT results [38]. In HIV-patients with FUO, controversy also remains whether a correlation exists between [^18^F]FDG uptake and HIV viremia [36, 56–58].

In conclusion, several pre-scan laboratory tests are routinely used depending on the clinical setting. However, their impact on [^18^F]FDG PET/CT yield is unclear and further studies are warranted especially aiming to establish a valid CRP cut-off in FUO (currently ranging from 7 to 50 mg/L). On the other hand, the yield or helpfulness of [^18^F]FDG PET/CT in the presence of normal or low CRP values is very low.

### Pre-scan imaging

Some relatively simple and inexpensive diagnostic tests are still recommended as first-line for patients with FUO/IUO to separate relatively more apparent diseases from true FUO/IUO cases, but there is no consensus on which modality to employ. Some sugge st abdominal ultrasound and chest X-ray in low-resource areas, some suggest CT, and others suggest an imaging approach guided by symptoms and clinico-biochemical findings, but there is no firm evidence to support a standardized imaging strategy that should routinely precede [^18^F]FDG PET/CT [29, 59, 60].

## Practical scan considerations

Generally, scan protocol and recommended administered [^18^F]FDG activity should adhere to existing EANM guidelines for tumour and infection imaging [61, 62].

### Patient preparation

[^18^F]FDG administration should be preceded by fasting for at least 6 h. In patients with suspected cardiac pathology (e.g. endocarditis, cardiac sarcoidosis) a prolonged fasting is recommended for at least 12–24 h, in combination with a high-fat low-carbohydrate diet to ensure optimal suppression of physiological myocardial uptake. Supplemental intravenous low-dose unfractionated heparin (50 IU/kg 15 min prior to [^18^F]FDG administration) may also be considered especially in patients unable to complete long-term fasting and diet, e.g. diabetics [63]. Plasma glucose levels are recommended to be < 200 mg/dL (11 mmol/L). Even though the effect of hyperglycemia on [^18^F]FDG uptake is probably less pronounced on inflammatory cells compared to malignant cells [64], a recent study showed that the yield of FDG PET/CT in bacteremia of unknown origin was lower in patients with moderate to severe hyperglycemia compared to normoglycemic patients [65].

Pre-scan treatment with corticosteroids (e.g. prednisone, prednisolone, methylprednisolone) is known to reduce uptake in inflammatory cells and hampers [^18^F]FDG PET/CT helpfulness in inflammatory conditions like vasculitis and polymyalgia rheumatic [66, 67]. Thus, corticosteroids may negatively influence the outcome of [^18^F]FDG PET/CT in FUO [34]. Treatment for longer than 3 days may significantly reduce [^18^F]FDG uptake in vasculitis [67], whereas 8 weeks treatment significantly reduced sensitivity in polymyalgia rheumatic [66]. In the latter study, one week of tapering followed by one week of discontinuation significantly increased sensitivity again but, generally speaking, there is no established lower limit in the literature. Corticosteroids should be reduced to a minimum or temporarily withdrawn, especially when vasculitis is suspected. Despite the few available results with doses of 15–60 mg [64, 68], a pragmatic recommendation is to postpone scans in patients treated with doses higher than 15 mg for > 3 days to reduce the risk of false-negative findings.

In contrast to glucocorticoids, the impact of antibiotic treatment is less well-known in the setting of FUO/IUO. Data from one study on [^18^F]FDG PET/CT in bloodstream infections reported a reduced possibility to find the focus of infection after more than one week of antibiotics with a further drop with increasing duration of treatment [69]. One study in FUO patients reported that empiric antibiotic therapy is associated with non-contributory [^18^F]FDG PET/CT [29], whereas others did not observe any association [38, 40, 70].

In conclusion, the effect of hyperglycemia is probably less pronounced than in cancer work-up, but plasma glucose levels are still recommended to be < 200 mg/dL (11 mmol/L) and thorough patient preparation is especially important if a cardiac focus is suspected. PET/CT is not recommended in patients under glucocorticoid treatment > 15 mg for more than 3 days, but scans can be performed despite pre-scan antibiotic treatment.

### Protocol

A major advantage of [^18^F]FDG PET/CT over CT, MRI and ultrasound is the possibility to screen the whole body in patients with suspected systemic disease and few diagnostic clues. The scan field should minimally include the vertex to mid-thighs, but a total-body acquisition could be considered.

Stand-alone PET is nowadays considered obsolete in FUO and only combined [^18^F]FDG PET/CT will be addressed in this document A disadvantage of hybrid-PET/CT over CT alone is the higher combined radiation exposure [71], which should be kept as low as possible especially in children and young adults [33]. Depending on the clinical indication, CT may be contrast-enhanced or non-enhanced [41]. The latter is often referred to as low-dose, although this may be misleading, and the actual settings are usually based on local practice more than guidelines. Adding CT to PET results in an additional exposure of approximately 2–10 mSv depending on protocols on top of the radiation exposure of FDG (0.019 mSv/MBq). In the literature on FUO, most studies used a non-enhanced “low-dose” CT [14, 18, 19, 29, 32, 33, 37, 38, 40, 44, 45, 48–50, 52, 54, 69, 72–74] while few studies were performed with contrast-enhanced CT [26, 39, 44, 48, 51, 69, 75]. However, head-to-head comparisons are lacking to determine the incremental value of upfront contrast-enhanced CT. Solid malignancies and abscesses are notable exceptions, and if these are suspected upfront, contrast-enhanced CT is indicated but may be performed in a separate setting.

In conclusion, a non-enhanced “low-dose” CT is sufficient in FUO/IUO to define the location of metabolic abnormalities that could lead to the diagnosis. There is usually limited value of contrast-enhanced CT because of the often relatively minor morphologic changes from infectious and inflammatory processes, especially at early stages.

### Interpretation and reporting

Interpretation and reporting is similar to [^18^F]FDG PET/CT scans for tumor imaging and non-oncological indications. The interpretation is primarily based on a visual assessment whereas only limited and heterogeneous data is available on semi-quantitative interpretation criteria [16, 20, 39, 76–78]. Findings may be focal (e.g. abscesses, endocarditis), diffuse (e.g. colitis, large vessel vasculitis), or confined by anatomic structures (e.g. spondylodiscitis, graft material). Some physiologic, reactive [^18^F]FDG-activity is often seen diffusely around implants but it is usually not difficult to distinguish this uptake from pathologic activity characterized by a heterogeneous/multifocal pattern. In patients with metallic or prosthetic implants, non-attenuation corrected PET images are helpful to distinguish true uptake from artefacts. Focal [^18^F]FDG uptake is more typical for infection than diffuse low-grade uptake. Finally, scans may display indirect signs of infection and inflammation, e.g. diffuse uptake in the bone marrow and spleen [16].

## Overall diagnostic yield

Due to the heterogeneity of potential diagnoses, a lack of well-defined reference standards, and many cases without a final diagnosis, the classical approach of determining the diagnostic performance of a test by reporting its sensitivity, specificity and predictive values may not be the most appropriate method. The majority of studies consider only true positive [^18^F]FDG PET foci as helpful, i.e. those that directly lead to a diagnosis. True negative findings are rarely included, even though patients with a negative [^18^F]FDG PET/CT usually are more likely to show a spontaneous clinical regression or remission [38, 44, 75, 79–81]. Secondly, true negative scans indicate a potential to reduce additional futile imaging, which contributes to the cost-effectiveness of [^18^F]FDG PET/CT when performed early in the diagnostic workup [3, 22]. On the other hand, studies also reported false positive findings in as many as 26–33% of patients that may lead to subsequent additional examinations [19, 35, 38, 43, 74, 82]. Importantly, a significant proportion of FUO/IUO patients (7–47%) remains without a final diagnosis after [^18^F]FDG PET/CT.

Even though many studies report sensitivities and specificities of [^18^F]FDG PET/CT in the context of FUO/IUO, the terminology of diagnostic yield or helpfulness is a better parameter, i.e. the proportion of all PET scans that help the clinicians in subsequent patient management [23, 27, 28].

Several meta-analyses have demonstrated the usefulness of [^18^F]FDG PET/CT in reaching the final diagnosis of FUO/IUO with a diagnostic yield of 56–60%, which is at least 30% higher than conventional CT, and a significantly better association between negative scans and spontaneous remission (Table 1) [3–6, 12, 22, 83]. Regarding the latter, a recent meta-analysis reported a significantly higher cumulative incidence of spontaneous remission of 20–78% in patients with negative [^18^F]FDG PET/CT results compared to 0–48% in those with positive results [6]. [^18^F]FDG PET/CT is also superior to [^18^F]FDG PET alone and other nuclear imaging methods such as [^67^Ga]Ga-citrate or leukocyte scintigraphy [22, 83]. Besides mere diagnostic yield, Besson et al. also reported that abnormal [^18^F]FDG uptake is associated with a statistically significant higher rate of definitive diagnoses compared to normal scans (83% vs. 36%) with a pooled odds ratio of 8.94 [3]. Other than the general FUO/IUO populations, two additional meta-analyses were identified in specific subpopulations. One on intensive care unit (ICU)-patients reported that [^18^F]FDG PET/CT is equally clinically helpful in the ICU-setting, i.e. in 65% of patients [84]. The one on paediatric patients did not assess diagnostic yield per se, but concluded that PET-positive patients were significantly more likely to receive a final diagnosis compared to normal scans (odds-ratio 17) [85].

**Table 1:** Systematic reviews/meta-analyses

Authors	Year	*N* (studies)	*N* (patients)	Sensitivity (95% CI)	Specificity (95% CI)	LR+ (95% CI)	LR- (95% CI)	Diagnostic yield/ clinical helpfulness
Besson et al. [3]	2016	14 (7 PET/CT)	712 (401 PET/CT)	*NR*	*NR*	*NR*	*NR*	0.58
Bharucha et al. [5]	2017	18	905	*NR*	*NR*	*NR*	*NR*	0.56
Dong et al. [22]	2011	4	174	0.98 (0.94-1.0)	0.86% (0.75–0.93)	5.8 (3.3–10)	0.05 (0.01–0.25)	0.60
Hao et al. [4]	2013	15 (1 pediatric)	595 (77 pediatrics)	0.85 (0.81–0.88)	*NR*	*NR*	*NR*	*NR*
Kan et al. [12]	2019	23	1927	0.84 (0.79–0.89)	0.63 (0.49–0.75)	2.3 (1.5–3.4)	0.25 (0.16–0.38)	0.59
Takeuchi et al. [83]	2016	22	1137	0.86 (0.81–0.90)	0.52 (0.36–0.67)	*NR*	*NR*	0.58
Takeuchi et al. [6]	2018	9	418	*NR*	*NR*	*NR*	*NR*	Negative scan associated with spontaneous remission (RR = 5.6, *p* < 0.001)
**Special populations**								
Huang et al. [84]	2020	4 (ICU patients)	87	0.94	0.66	*NR*	*NR*	0.65
Li et al. [85]	2022	6 (pediatric)	191	*NR*	*NR*	*NR*	*NR*	Abnormal scans had OR 17 to achieve definite diagnoses compared to normal scans

CI: confidence interval; LR+: positive likelihood ratio; LR-: negative likelihood ratio; NR: not reported; RR: risk ratio; OR: odds ratio

The literature search identified 49 relevant clinical studies on the diagnostic value of [^18^F]FDG PET/CT in adult patients with FUO/IUO [13–20, 26, 29, 32, 34, 35, 37–39, 43–52, 54, 55, 72, 74–82, 86–96]; an overview is presented in Table 2. Most of the clinical studies were retrospective single centre cohorts (*n* = 34, 69%) with large variation in sample size (12–524 cases). Most studies included only patients with FUO (*n* = 30, 61%), while fourteen studies included patients with a case-mix of FUO/IUO (29%), and five studies evaluated only patients with IUO (10%). All included studies focused on diagnostic or clinical value of [^18^F]FDG PET/CT in FUO or IUO patients, but two studies also investigated cost-effectiveness [76, 86], and two studies compared [^18^F]FDG PET/CT with [^67^Ga]Ga-citrate scintigraphy [81, 90]. Final diagnosis was related to infection in 10–68% patients, NIID in 8–60% patients, malignancy in 3–36% patients and a variety of uncommon conditions in 2–31% patients. Helpfulness of [^18^F]FDG PET/CT in the diagnosis or management of FUO ranged from 19 to 96% with higher values when both true positives and true negatives were considered. In some studies, [^18^F]FDG PET/CT was deemed essential in 6–26% of patients to reach a final diagnosis [26, 29, 32, 37, 75, 78] because no other investigation, including CT of chest and abdomen, was able to establish a diagnosis. With the highly variable populations and sample sizes in the available literature, there is a significant risk of selection bias.

**Table 2:** Original studies (adults only)

Authors	Year	*N*	Population	Study Design	Final diagnosis	Yield	Clinical helpfulness	Essential	Sensitivity	Specificity	PPV	NPV
Abdelrahman et al. [39]	2018	27	FUO	*P*	0.92	0.85 (TP)	NR	NR	0.95	0.67	0.96	0.67
Akyüz et al. [47]	2023	132	IUO	R	0.56	0.74 (PET+)	0.36	NR	NR	NR	NR	NR
Balink et al. [55]	2009	68	FUO	R	0.65	0.56 (TP)	0.56	NR	1.00	0.90	0.93	1.00
Balink et al. [44]	2014	140	IUO	R, M	0.74	0.68 (TP)	0.51	NR	0.94	0.83	0.93	0.77
Balink et al. [13]	2015	498	FUO/IUO	R, M	0.66	0.59 (TP)	NR	NR	0.89	0.89	0.94	0.80
Becerra Nakayo et al. [86]	2012	20	FUO	R	NR	0.55 (TP)	NR	NR	0.78	0.83	0.92	0.62
Betrains et al. § [17]	2023	439 (187)	FUO/IUO	R	0.64	0.49 (TP)	0.25	NR	0.93	0.35	NR	NR
Bilici et al. [49]	2021	97	IUO	*P*	0.90	0.60 (TP)	0.61	NR	0.67	1.0	1.0	0.26
Bouter et al. [14]	2016	72	IUO/fever	R	0.83	0.65 (TP)	NR	NR	0.81	0.86	NR	NR
Buch-Olsen et al. [87]	2014	57	FUO	R	0.91	NR	0.75 (TP + TN)	NR	NR	NR	NR	NR
Buchrits et al. [26]	2021	303	FUO	R	0.72	0.33 (TP)	NR	0.26	0.89	0.81	NR	NR
Chen et al. [76]	2022	524	FUO	*P*	0.91	0.91 (PET+)	NR	NR	NR	NR	NR	NR
Chen et al. [88]	2022	326	FUO/IUO	R	0.91	0.95 (PET+)	0.96	NR	NR	NR	NR	NR
Crouzet et al. [29]	2012	79	FUO	R	0.77	0.57 (TP)	0.19	0.25	0.98	0.87	NR	NR
Ergül et al. [82]	2011	24	FUO	R	0.54	0.50 (TP)	0.63	NR	0.92	0.45	0.63	1.0
Eynath et al. §§ [75]	2023	303	FUO	R	0.72	0.33 (TP)	NR	0.26	0.89	0.81	NR	NR
Federici et al.* [32]	2010	14	FUO/IUO	R	0.71	0.50 (TP)	0.50	0.23	0.70	0.75	0.88	0.5
Ferda et al. [77]	2010	48	FUO	R	0.92	0.96 (TP)	0.77	NR	0.97	0.75	NR	NR
Gafter-Gvili et al. [89]	2015	112	FUO	R	0.74	0.46 (TP)	0.66	NR	0.72	0.58	0.74	0.54
Garcia-Vicente et al. [38]	2018	67	FUO	R	0.88	0.78 (PET+)	0.52	NR	0.84	0.31	NR	NR
Georga et al. [72]	2020	50	FUO	R	0.86	0.84 (PET+)	0.70	NR	0.95	0.50	0.85	0.75
Holubar et al. [46]	2022	317	IUO	R	0.72	0.50 (TP)	0.75 (TP + TN)	NR	0.84	0.62	0.77	0.72
Hung et al.# [90]	2017	58	FUO	*P*	0.79	0.57 (TP)	0.72	NR	0.79	0.56	0.83	0.50
Jaruskova et al. [91]	2006	94	FUO/fever	R	NR	0.46 (PET+)	0.36	NR	NR	NR	NR	NR
Kei et al. [92]	2010	12	FUO	R	0.58	0.42 (TP)	0.42	NR	NR	NR	NR	NR
Keidar et al. [80]	2008	48	FUO	*P*	0.60	0.56 (PET+)	0.90 (TP + TN)	NR	1.00	0.81	0.81	1.00
Kim et al. [35]	2012	48	FUO	R	0.85	0.85 (PET+)	0.56	NR	0.92	0.23	NR	NR
Knappe et al. [51]	2023	130	IUO	R	0.76	0.65 (TP)	NR	NR	0.93	0.93	0.97	0.86
Kubota et al. [34]	2011	81	FUO	R, M	0.75	0.52 (TP)	0.54	NR	0.81	0.75	NR	NR
Kubota et al.# [81]	2021	128	FUO	P, M	0.72	0.33 (TP)	0.33	NR	0.45	0.40	0.67	NR
Letertre et al. [43]	2021	44	FUO	R	0.70	NR	0.44	NR	0.85	0.37	0.58	0.70
Ly et al. [18]	2022	103	FUO/IUO	P, M	0.56	0.29 (PET+)	0.19	NR	0.36	0.81	NR	NR
Mahajna et al. [48]	2021	128	FUO	R	0.74	0.68 (PET+)	0.48	NR	0.70	0.37	0.70	0.37
Manohar et al. [93]	2013	103	FUO	R	0.67	0.61 (PET+)	0.60	NR	0.90	0.97	0.98	0.83
Mulders-Manders et al. [45]	2021	104	FUO/IUO	R	0.65	NR	0.45	NR	NR	NR	NR	NR
Ogut et al. [20]	2022	58	FUO/IUO	R	0.90	0.64 (TP)	0.72 (TP + TN)	NR	0.88	0.37	0.79	0,55
Okuyucu et al. [50]	2018	76	FUO	R	0.85	0.62 (TP)	0.74 (TP + TN)	NR	0.75	0.69	0.92	0.38
Pedersen et al.* [94]	2012	22	FUO	R	0.60	0.45 (TP)	0.83	NR	0.67	0.71	0.83	0.50
Pelosi et al.* [79]	2011	24	FUO	R	0.71	0.46 (TP)	0.87 (TP + TN)	NR	0.50	0.50	0.85	0.85
Pereira et al. [37]	2016	76	FUO	R	0.93	0.74 (PET+)	0.61	0.17	0.77	0.31	0.61	0.50
Schönau et al. [19]	2018	240	FUO/IUO	*P*	0.79	0.57 (TP)	0.57	NR	0.91	0.22	0.65	0.62
Sheng et al. [95]	2011	48	FUO	R	0.75	0.83 (PET+)	0.67	NR	0.89	0.33	0.80	0.50
Singh et al. [78]	2015	47	FUO	*P*	0.53	0.74 (PET+)	0.38	0.06	NR	NR	0.51	NR
Tokmak et al. [96]	2014	25	FUO	*P*	0.84	0.60 (TP)	0.84 (TP + TN)	NR	0.94	0.80	NR	NR
Tsuzuki et al. [54]	2021	50	FUO/IUO	R	0.74	0.58 (TP)	0.66 (TP + TN)	NR	NR	NR	NR	NR
Wang et al. [16]	2019	376	FUO/IUO	R, M	0.91	0.95 (PET+)	0.90	NR	NR	NR	NR	NR
Wang et al. [15]	2020	253	FUO/IUO	*P*	0.88	0.58 (PET+)	NR	NR	0.88	0.15	0.59	0.47
Weitzer et al. [52]	2022	300	FUO/IUO	R	0.84	0.54 (TP)	0.83 (TP + TN)	NR	0.80	0.90	NR	NR
Zhu et al. [74]	2020	89	FUO	R	0.74	0.55 (TP)	0.55	NR	0.85	0.26	NR	NR

FUO: fever or unknown origin; IUO: inflammation of unknown origin; NPV: negative predictive value; NR: not reported; P: prospective; PPV: positive predictive value; R: retrospective; M: multicentre; TP: true positive; ^#^[^18^F]FDG PET/CT vs. [^67^Ga]Ga-citrate comparison; §Only a subset underwent PET/CT (187/439, 43%), all numbers are for the entire cohort

§§ Based on the same study as Buchrits et al.

Balink et al. performed the largest multicentre retrospective study on FUO/IUO patients (*n* = 498) [13]. [^18^F]FDG PET/CT had a high diagnostic accuracy (89%) and the addition of [^18^F]FDG PET/CT to a model for the prediction of a diagnosis including CRP, ESR and age resulted in a significant change in patient classification in 42% of patients.

The largest prospective study (*n* = 524) on the diagnostic value of [^18^F]FDG PET/CT in patients with FUO aimed to develop a diagnostic model to distinguish the different causes of FUO [76]. [^18^F]FDG PET/CT showed positive findings in 477 (91%) patients (diffuse or focal high uptake in various organs and tissues). The diagnostic model including [^18^F]FDG PET/CT and relevant clinical parameters (e.g. blood cell counts, inflammatory and immunological indicators, age) showed a good performance in discriminating the cause of FUO with AUCs for infection, malignancy, and NIID of 0.88, 0.93 and 0.95, respectively. Previous studies have also divided positive [^18^F]FDG PET/CT studies into focal and nonspecific abnormal uptake [15, 16]. The latter is represented by diffuse high [^18^F]FDG uptake of spleen and bone marrow and multiple lymph nodes with high [^18^F]FDG uptake and symmetrical distribution. This pattern was considered non-contributing to the diagnosis or even false positives in other studies [16, 19, 80, 82, 89]. However, nonspecific abnormal [^18^F]FDG uptake as well as a negative [^18^F]FDG PET/CT scan could also be of benefit for the patients.

The variable performance of [^18^F]FDG PET/CT in FUO/IUO may be explained, as previously described, by a multitude of factors; the complexity of patients, definitions of FUO/IUO, retrospective and observational study designs, relatively small sample sizes, differences in pre-scan work-up, differences in [^18^F]FDG PET/CT timing, and varying definitions of a clinical helpful result.

Overall, the results of most studies show that a positive [^18^F]FDG PET/CT is often contributive and, in some cases, is essential to establish a diagnosis by identifying potential causes of FUO/IUO, localizing sites for further evaluation, and guiding further management. Conversely, a negative scan excludes focal disease and predicts a favourable prognosis.

## Diagnostic yield in specific populations

As mentioned above, [^18^F]FDG PET/CT provides a good diagnostic yield in the general population of adults with FUO/IUO and can be considered as a primary imaging tool. In addition to the general adult population, [^18^F]FDG PET/CT has also been evaluated in specific subpopulations with FUO/IUO, including children, intensive care unit patients, patients with end stage renal disease, and HIV (Table 3).

**Table 3:** Original studies, special populations

Authors	Year	Population	*N*	Study design	Sensitivity	Specificity	PPV	NPV	Helpful
Nygaard et al. [97]	2022	Children	35	R	*NR*	*NR*	*NR*	*NR*	0.77
Pijl et al. [41]	2020	Children	110	R	0.86	0.79	0.84	0.81	0.53
Chang et al. [98]	2016	Children	19	R	0.88	0.67	0.93	0.50	0.74
Yang et al. [99]	2015	Children	5	R	*NR*	*NR*	*NR*	*NR*	0.40
Blokhuis et al. [100]	2014	Children	28 (FUO) 11 (IUO)	R	0.80 0.78	0.78 0.67	0.67 0.88	0.88 0.50	0.32 0.55
Jasper et al. [33]	2010	Children	30	R	*NR*	*NR*	*NR*	*NR*	0.53
Simons et al. [101]	2009	ICU	33	*P*	1.0	0.79	0.84	1.0	0.60
Lawal et al. [40]	2019	End-stage renal disease	46	R	*NR*	*NR*	*NR*	*NR*	0.48
Tek Chand et al. [102]	2017	End-stage renal disease	20	R	*NR*	*NR*	*NR*	*NR*	0.95
Martin et al. [36]	2013	HIV	20	*P*	*NR*	*NR*	*NR*	*NR*	0.80
Castaigne et al. [103]	2009	HIV	10	R	*NR*	*NR*	*NR*	*NR*	0.90

N: number; PPV: positive predictive value; NPV: negative predictive value; R: retrospective; NR: not reported; P: prospective; FUO: fever of unknown origin; IUO: inflammation of unknown origin; ICU: intensive care unit; HIV: human immunodeficiency virus

### Children with FUO/IUO

Six retrospective studies (*n* = 238 patients) evaluated the value of [^18^F]FDG PET/CT in children with FUO/IUO, including one study in pediatric transplant patients [33, 41, 97–100]. The largest study by Pijl et al. including 110 children with FUO showed that [^18^F]FDG PET/CT identified a true positive cause of fever in 53 (48%) children, and in 58 children (53%) treatment modifications were based on PET/CT results. The sensitivity and specificity were 86% and 79%, respectively, and CRP levels were positively associated with finding a true focus [41]. [^18^F]FDG PET/CT was considered helpful in 48%, whereas 38% remained without a final diagnosis after [^18^F]FDG PET/CT [33, 98, 100]. In addition and similar to the adult population, the same issues were encountered regarding different definitions of helpful and whether or not negative scans were considered helpful, non-contributory or unclassified [33, 97, 100]. The clinical helpfulness across the six studies had a considerable span but with sample sizes ranging from 5 to 110 patients, the relative weight of these results are different and not readily comparable.

### Patients in the ICU

Only one prospective study on the use of [^18^F]FDG PET/CT in critically ill, mechanically ventilated patients admitted to the ICU with FUO/IUO is available. The study reported that 21/35 scans (60%) were true positive, 11/35 scans were true negative, and three scans were considered false positive resulting in a sensitivity of 100%, specificity of 79% and overall accuracy of 91%. The authors emphasized that, besides the high accuracy, a normal [^18^F]FDG PET/CT scan was also considered important, since it ruled out infections requiring prolonged antibiotic therapy or drainage [101].

### Patients with end-stage renal disease

Two retrospective studies evaluated the role of [^18^F]FDG PET/CT in FUO patients suffering from end-stage renal disease on renal replacement therapy. The first study evaluating 20 patients (18 on hemodialysis, two on peritoneal dialysis) showed active lesions in 15 patients, and negative scan results in five patients. In all but one patient, imaging results led to a change in treatment [102].

In the second study, 46 patients underwent [^18^F]FDG PET/CT (21 on hemodialysis, 8 on peritoneal dialysis, and 17 renal transplants). Twenty-nine scans showed at least one focus of increased uptake, 17 scans turned out to be negative and [^18^F]FDG PET/CT was helpful in identifying the cause of FUO in 22 patients (48%). CRP and procalcitonin levels were significantly higher in patients with a helpful [^18^F]FDG PET/CT scan [40].

### Patients with HIV

One study prospectively evaluated the performance of [^18^F]FDG PET/CT in 20 HIV-infected patients with FUO in comparison to 10 HIV-infected high viraemic patients without FUO. Despite the limited number of included patients, high viraemic status did not interfere with a correct scan interpretation but different uptake patterns were observed: more peripheral active lymph nodes in patients without FUO compared to more central active lymph nodes in FUO patients. [^18^F]FDG PET/CT contributed to the diagnosis or exclusion of a focal etiology in 80% of the patients. The absence of active central lymph nodes in FUO patients showed a 100% negative predictive value for focal disease. Lymph node biopsy in central active areas allowed identification of underlying disease in all patients with FUO. Furthermore, [^18^F]FDG PET/CT demonstrated more extensive disease than conventional imaging [36]. The other study in this patient population found [^18^F]FDG PET/CT helpful in 9/10 patients, six with infection and three with malignancy [103].

In conclusion, [^18^F]FDG PET/CT can be considered useful for evaluating the cause of FUO/IUO in these specific populations, taking into account the same caveats as the general adult FUO/IUO population. The diagnostic yield and helpfulness of [^18^F]FDG PET/CT in these specific patient groups are similar to the results in the general adult population.

## Impact on patient management and prognosis

A correct and early diagnosis can change the therapeutic strategy, i.e. initiate new therapy, modulate already instituted therapy, or switch therapy regimen completely. Moreover, it may guide specific invasive and noninvasive procedures such as biopsy or drainage, specific serology, or cultures of blood, urine, or tissues with a direct impact also on costs [55].

A major contribution of [^18^F]FDG PET/CT for obtaining the final histological diagnosis is to guide the clinician to the most appropriate and accessible site for biopsy to confirm the underlying etiology [72].

An indirect impact is to exclude several causes and to narrow down the range of possible diagnoses. About 43–75% of adult patients with undiagnosed FUO experience spontaneous remission of fever before reaching a final diagnosis [7, 104], and empirical treatment with corticosteroids or nonsteroidal anti-inflammatory drugs does not affect this rate. Identifying predictors of spontaneous resolution could reduce the use of unnecessary, invasive tests or empirical treatments, and furthermore potentially reduce patient anxiety. As such, a negative PET/CT result can be a good predictor of favorable prognosis in patients with undiagnosed FUO after a series of unsuccessful investigations; watchful waiting may be a valid option for undiagnosed FUO patients with no specific findings. One systematic review studied the association of [^18^F]FDG PET/CT results with spontaneous remission in FUO. Patients with negative scan results were significantly more likely to have spontaneous remission of fever than were those with positive scan results, with a summary risk ratio of 5.6 (95% CI, 3.4–9.2) [6]. This may also have a direct impact on patient outcome, as prognosis was generally good and mortality very low in patients in whom a final diagnosis could not be obtained [105–107].

As FUO is caused by a wide variety of diseases; the overall prognosis highly depends on the underlying disease [8], with malignancy probably having the most prominent association with mortality [108–110].

However, although promising, more robust evidence is required to evaluate the prognostic role of [^18^F]FDG PET/CT in FUO/IUO patients.

## Cost-effectiveness

An important aspect of any imaging strategy is cost, and to date, few studies assessed the cost-effectiveness of [^18^F]FDG PET/CT in FUO/ IUO reporting that [^18^F]FDG PET/CT is cost-effective in specific scenarios of FUO/IUO. Beccera-Nakayo et al. included 20 FUO-patients that underwent a scan after a mean hospital stay of 28 days. They registered all costs in the FUO-process including hospitalization and diagnostic procedures prior to PET/CT. They found a potential cost reduction of 5,471 € (49%) if [^18^F]FDG PET/CT was performed earlier [86]. Balink et al. found similar results in 92 IUO-patients, 46 who did not undergo [^18^F]FDG PET/CT (group A) and 46 who underwent [^18^F]FDG PET/CT (group B). Costs in group B were reduced with 5.298 € (42%) compared to group A, and at the same time a definite diagnosis was reached in more than twice the patients, i.e. 32/46 in group B versus 14/46 in group A [111]. In the largest and most recent study, Chen et al. included 741 FUO/IUO-patients; 44% underwent [^18^F]FDG PET/CT They did find higher overall costs, more additional tests and longer hospitalization in the [^18^F]FDG PET/CT-group compared to controls. However, this population was older and had more critically ill patients, and again the number of definite diagnosis was significantly higher in the intervention group. Furthermore, the total costs and hospitalization stay was reduced if [^18^F]FDG PET/CT was performed within 7 days [88].

## Recommendations

**Fig. 1:**
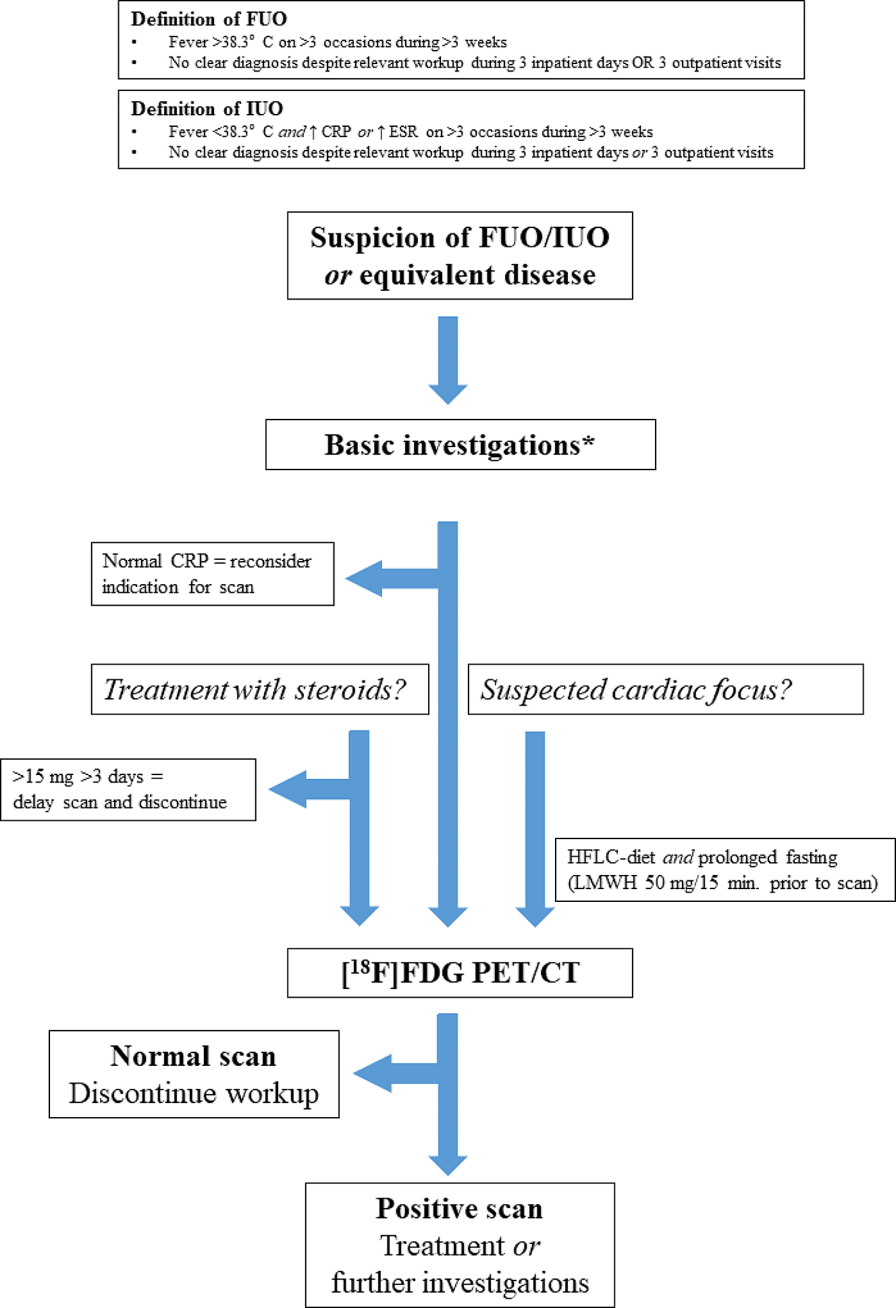
Suggested flowchart for the diagnostic strategy of fever of unknown origin (FUO), inflammation of unknown origin (IUO), and equivalent cases with nonspecific suspicion systemic infection or inflammation. *As discussed in the section on pre-scan work-up, there is no consensus on basic investigations. Most recommend at least some inflammatory markers, but any subsequent step(s) are often left at the discretion of the treating physician and may be as much based on local tradition as on evidence FUO: fever of unknown origin; IUO: inflammation of unknown origin; CRP: c-reactive protein; ESR: erythrocyte sedimentation rate; WBC: white blood cells; CXR: chest x-ray; HFLC: high-fat low-carbohydrate; LMWH: low molecular-weight heparin

Based on the above-mentioned evidence from the published literature, several recommendations are summarized in Table 4 and we have developed a flowchart shown in Fig. 1 with a suggested pathway when to use FDG PET/CT in the setting of FUO/IUO according to literature evidence and expert opinion.

**Table 4:** Summary of conclusions and recommendations

**General**
FUO and IUO are overlapping entities with strict definitions, but not all patients meet these stringent criteria; suspicion of systemic infection or inflammation is a valid indication for [^18^F]FDG PET/CT equivalent to true FUO/IUO
Literature on [^18^F]FDG PET/CT in FUO/IUO is heterogeneous and primarily based on limited retrospective studies
White blood cell scintigraphy, [^67^Ga]Ga-citrate scintigraphy, and stand-alone [^18^F]FDG PET are obsolete in FUO/IUO

**Pre-scan considerations**
There is no consensus in literature on preparatory laboratory investigations or imaging. CRP, ESR, white blood cell count, and chest x-ray/abdominal ultrasound (based on clinical/biochemical findings) are minimum requirements prior to [^18^F]FDG PET/CT – clinical suspicion may guide further choices
There are no established cut-off values for CRP, but when CRP levels are normal the indication for [^18^F]FDG PET/CT should be reconsidered as the yield or helpfulness of [^18^F]FDG-PET/CT in the presence of normal or low CRP values is very low.
High-fat low-carbohydrate diet, prolonged fasting, with or without heparin administration 15 min before the FDG injection is essential to suppress physiological myocardial uptake in suspected cardiac infections
Pre-scan antibiotic treatment (< 7 days) has probably little effect on diagnostic yield
Pre-scan corticosteroids > 15 mg for more than 3 days may hamper uptake in inflammatory cells and should ideally be started after scan (or discontinued prior to scan)

**Scan considerations**
Acquisition from vertex to mid-thigh is recommended, unless more distal foci are suspected
Low-dose CT without contrast-enhancement is sufficient in most cases

**Diagnostic yield**
Diagnostic yield varies widely dependent on the populations and interpretation criteria, but [^18^F]FDG PET/CT is generally considered to be helpful in 50–60%
Positive findings (true positive) can directly guide treatment or indirectly guide subsequent diagnostic procedures or tests
Negative scans (true negative) can be considered as helpful as positive findings; spontaneous remission is significantly more likely in patients with a negative scan
[^18^F]FDG PET/CT is equally useful in suspected FUO/IUO in specific populations, e.g. pediatric patients, intensive care patients, end-stage renal disease, and HIV

FUO: fever of unknown origin; IUO: inflammation of unknown origin; CRP: c-reactive protein; ESR: erythrocyte sedimentation rate; HIV: human immunodeficiency virus

## Conclusions

FUO/IUO remains a clinical challenge due to the heterogeneous patient presentation, a wide variety of differential diagnoses, and the lack of an established workup strategy. The literature is as challenging and heterogeneous as the population, which hampers pooling of data and direct comparison between studies.

However, [^18^F]FDG PET/CT has a definite role in the diagnostic workup with an overall diagnostic yield/helpfulness of 50–60%. A positive scan is often contributory and, in some cases, even essential to diagnosis, whereas a negative scan may be equally important as it excludes focal disease and predicts a favorable prognosis. Similar results are obtained in specific populations such as ICU-patients, children and HIV-patients.

### Electronic supplementary material

Below is the link to the electronic supplementary material.

Supplementary Material 1

Supplementary Material 2

Supplementary Material 3

## Data Availability

All data generated or analysed during this study are included in this published article [and its supplementary information files].

## References

[1] Gormsen LC, Hess S (2020). Challenging but clinically useful: Fluorodeoxyglucose PET/Computed tomography in Inflammatory and Infectious diseases. PET Clin.

[2] Treglia G (2019). Diagnostic performance of (18)F-FDG PET/CT in Infectious and Inflammatory diseases according to published Meta-analyses. Contrast Media Mol Imaging.

[3] Besson FL, Chaumet-Riffaud P, Playe M, Noel N, Lambotte O, Goujard C (2016). Contribution of (18)F-FDG PET in the diagnostic assessment of fever of unknown origin (FUO): a stratification-based meta-analysis. Eur J Nucl Med Mol Imaging.

[4] Hao R, Yuan L, Kan Y, Li C, Yang J (2013). Diagnostic performance of 18F-FDG PET/CT in patients with fever of unknown origin: a meta-analysis. Nucl Med Commun.

[5] Bharucha T, Rutherford A, Skeoch S, Alavi A, Brown M, Galloway J (2017). Diagnostic yield of FDG-PET/CT in fever of unknown origin: a systematic review, meta-analysis, and Delphi exercise. Clin Radiol.

[6] Takeuchi M, Nihashi T, Gafter-Gvili A, Garcia-Gomez FJ, Andres E, Blockmans D (2018). Association of 18F-FDG PET or PET/CT results with spontaneous remission in classic fever of unknown origin: a systematic review and meta-analysis. Medicine.

[7] Bleeker-Rovers CP, Vos FJ, de Kleijn E, Mudde AH, Dofferhoff TSM, Richter C (2007). A prospective multicenter study on fever of unknown origin: the yield of a structured diagnostic protocol. Medicine.

[8] Mulders-Manders C, Simon A, Bleeker-Rovers C (2015). Fever of unknown origin. Clin Med.

[9] Blockmans D, Knockaert D, Maes A, De Caestecker J, Stroobants S, Bobbaers H (2001). Clinical value of [(18)F]fluoro-deoxyglucose positron emission tomography for patients with fever of unknown origin. Clin Infect Diseases: Official Publication Infect Dis Soc Am.

[10] Petersdorf RG, Beeson PB (1961). Fever of unexplained origin: report on 100 cases. Medicine.

[11] Durack DT, Street AC (1991). Fever of unknown origin–reexamined and redefined. Curr Clin Top Infect Dis.

[12] Kan Y, Wang W, Liu J, Yang J, Wang Z (2019). Contribution of 18F-FDG PET/CT in a case-mix of fever of unknown origin and inflammation of unknown origin: a meta-analysis. Acta Radiol.

[13] Balink H, Veeger NJ, Bennink RJ, Slart RH, Holleman F, van Eck-Smit BL (2015). The predictive value of C-reactive protein and erythrocyte sedimentation rate for 18F-FDG PET/CT outcome in patients with fever and inflammation of unknown origin. Nucl Med Commun.

[14] Bouter C, Braune I, Meller B, Sahlmann C, Ritter C, Meller J (2016). (18)F-FDG-PET/CT in unexplained elevated inflammatory markers. Join Entities Nuklearmedizin.

[15] Wang WX, Cheng ZT, Zhu JL, Xing MY, Zheng CF, Wang SJ (2020). Combined clinical parameters improve the diagnostic efficacy of (18)F-FDG PET/CT in patients with fever of unknown origin (FUO) and inflammation of unknown origin (IUO): a prospective study in China. Int J Infect Diseases: IJID: Official Publication Int Soc Infect Dis.

[16] Wang Q, Li YM, Li Y, Hua FC, Wang QS, Zhang XL (2019). 18)F-FDGPET/CT in fever of unknown origin and inflammation of unknown origin: a Chinese multi-center study. Eur J Nucl Med Mol Imaging.

[17] Betrains A, Boeckxstaens L, Moreel L, Wright WF, Blockmans D, Van Laere K (2023). Higher diagnostic yield of 18F-FDG PET in inflammation of unknown origin compared to fever of unknown origin. Eur J Intern Med.

[18] Ly KH, Costedoat-Chalumeau N, Liozon E, Dumonteil S, Ducroix JP, Sailler L et al. Diagnostic value of 18F-FDG PET/CT vs. chest-abdomen-pelvis CT scan in management of patients with fever of unknown origin, inflammation of unknown origin or episodic fever of unknown origin: a comparative multicentre prospective study. J Clin Med. 2022;11(2). 10.3390/jcm11020386 PMC8779072 35054081

[19] Schonau V, Vogel K, Englbrecht M, Wacker J, Schmidt D, Manger B (2018). The value of (18)F-FDG-PET/CT in identifying the cause of fever of unknown origin (FUO) and inflammation of unknown origin (IUO): data from a prospective study. Ann Rheum Dis.

[20] Öğüt TS, Erbasan F, Terzioğlu ME, Tazegul G, Yazısız V (2022). The Diagnostic Value of Fluoro-18 fluorodeoxyglucose (F-18 FDG) PET/CT in fever or inflammation of unknown origin: a retrospective study at a Rheumatology Clinic. Cureus.

[21] Vanderschueren S, Del Biondo E, Ruttens D, Van Boxelaer I, Wauters E, Knockaert DD (2009). Inflammation of unknown origin versus fever of unknown origin: two of a kind. Eur J Intern Med.

[22] Dong MJ, Zhao K, Liu ZF, Wang GL, Yang SY, Zhou GJ (2011). A meta-analysis of the value of fluorodeoxyglucose-PET/PET-CT in the evaluation of fever of unknown origin. Eur J Radiol.

[23] Hess S. FDG-PET/CT in fever of unknown origin, bacteremia, and febrile neutropenia. PET Clin. 2020;15(2):(in press). 10.1016/j.cpet.2019.11.002 32145888

[24] Kaya A, Ergul N, Kaya SY, Kilic F, Yilmaz MH, Besirli K (2013). The management and the diagnosis of fever of unknown origin. Expert Rev anti-infective Therapy.

[25] Kouijzer IJE, Mulders-Manders CM, Bleeker-Rovers CP, Oyen WJG. Fever of unknown origin: the value of FDG-PET/CT. Seminars in nuclear medicine. 2018;48(2):100–7. 10.1053/j.semnuclmed.2017.11.004 29452615

[26] Buchrits S, Gafter-Gvili A, Eynath Y, Bernstine H, Guz D, Avni T (2021). The yield of F(18) FDG PET-CT for the investigation of fever of unknown origin, compared with diagnostic CT. Eur J Intern Med.

[27] Wright WF, Kandiah S, Brady R, Shulkin BL, Palestro CJ, Jain SK (2024). Nuclear Medicine Imaging Tools in Fever of unknown origin (FUO): time for a Revisit and Appropriate Use Criteria.

[28] van Rijsewijk ND, FFA IJ, Wouthuyzen-Bakker M, Glaudemans A (2023). Molecular Imaging of Fever of unknown origin: an update. Semin Nucl Med.

[29] Crouzet J, Boudousq V, Lechiche C, Pouget JP, Kotzki PO, Collombier L (2012). Place of (18)F-FDG-PET with computed tomography in the diagnostic algorithm of patients with fever of unknown origin. Eur J Clin Microbiol Infect Diseases: Official Publication Eur Soc Clin Microbiol.

[30] Lorenzen J, Buchert R, Bohuslavizki KH (2001). Value of FDG PET in patients with fever of unknown origin. Nucl Med Commun.

[31] Buysschaert I, Vanderschueren S, Blockmans D, Mortelmans L, Knockaert D (2004). Contribution of (18)fluoro-deoxyglucose positron emission tomography to the work-up of patients with fever of unknown origin. Eur J Intern Med.

[32] Federici L, Blondet C, Imperiale A, Sibilia J, Pasquali JL, Pflumio F (2010). Value of (18)F-FDG-PET/CT in patients with fever of unknown origin and unexplained prolonged inflammatory syndrome: a single centre analysis experience. Int J Clin Pract.

[33] Jasper N, Dabritz J, Frosch M, Loeffler M, Weckesser M, Foell D (2010). Diagnostic value of [(18)F]-FDG PET/CT in children with fever of unknown origin or unexplained signs of inflammation. Eur J Nucl Med Mol Imaging.

[34] Kubota K, Nakamoto Y, Tamaki N, Kanegae K, Fukuda H, Kaneda T (2011). FDG-PET for the diagnosis of fever of unknown origin: a Japanese multi-center study. Ann Nucl Med.

[35] Kim YJ, Kim SI, Hong KW, Kang MW (2012). Diagnostic value of 18F-FDG PET/CT in patients with fever of unknown origin. Intern Med J.

[36] Martin C, Castaigne C, Tondeur M, Flamen P, De Wit S (2013). Role and interpretation of fluorodeoxyglucose-positron emission tomography/computed tomography in HIV-infected patients with fever of unknown origin: a prospective study. HIV Med.

[37] Pereira AM, Husmann L, Sah BR, Battegay E, Franzen D (2016). Determinants of diagnostic performance of 18F-FDG PET/CT in patients with fever of unknown origin. Nucl Med Commun.

[38] Garcia-Vicente AM, Tello-Galan MJ, Amo-Salas M, Ros-Izquierdo J, Jimenez-Londono GA, La Rosa Salas B (2018). Do clinical and laboratory variables have any impact on the diagnostic performance of 18F-FDG PET/CT in patients with fever of unknown origin?. Ann Nucl Med.

[39] Abdelrahman SFE, El-nasr SD, Gadalla SIS (2018). E. H. Value of 18-F-FDG PET/CT in assessment of patients with fever of unknown origin. Egypt J Radiol Nuclear Med.

[40] Lawal IO, Popoola GO, Lengana T, Ankrah AO, Ebenhan T, Sathekge MM (2019). Diagnostic utility of (18)F-FDG PET/CT in fever of unknown origin among patients with end-stage renal disease treated with renal replacement therapy. Hell J Nucl Med.

[41] Pijl JP, Kwee TC, Legger GE, Peters HJH, Armbrust W, Schölvinck EH (2020). Role of FDG-PET/CT in children with fever of unknown origin. Eur J Nucl Med Mol Imaging.

[42] Yadav BK, Pannu AK, Kumar R, Rohilla M, Kumari S (2021). Fever of unknown origin in older adults: a prospective observational study from North India. J Assoc Physicians India.

[43] Letertre S, Fesler P, Zerkowski L, Picot MC, Ribstein J, Guilpain P et al. Place of the (18)F-FDG-PET/CT in the diagnostic workup in patients with classical fever of unknown origin (FUO). J Clin Med. 2021;10(17). 10.3390/jcm10173831 PMC8432230 34501277

[44] Balink H, Bennink RJ, Veeger NJ, van Eck-Smit BL, Verberne HJ (2014). Diagnostic utility of (18)F-FDG PET/CT in inflammation of unknown origin. Clin Nucl Med.

[45] Mulders-Manders CM, Kouijzer IJ, Janssen MJ, Oyen WJ, Simon A, Bleeker-Rovers CP (2021). Optimal use of [18F]FDG-PET/CT in patients with fever or inflammation of unknown origin. The quarterly journal of nuclear medicine and molecular imaging: official publication of the Italian Association of Nuclear Medicine (AIMN) [and] the International Association of Radiopharmacology (IAR). [and] Sect So.

[46] Holubar J, Broner J, Arnaud E, Hallé O, Mura T, Chambert B (2022). Diagnostic performance of (18) F-FDG-PET/CT in inflammation of unknown origin: a clinical series of 317 patients. J Intern Med.

[47] Akyüz Dağlı P, Güven SC, Coşkun N, Karakaş Ö, Armağan B, Orhan K (2023). Rheumatology experience with FDG PET / CT in inflammation of unknown origin: a single - centre report for determining factors associated with diagnostic precision. Clin Rheumatol.

[48] Mahajna H, Vaknin K, Ben Shimol J, Watad A, Abu-Much A, Mahroum N et al. The utility of 18FDG-PET/CT in diagnosing fever of unknown origin: the experience of a large tertiary medical center. Int J Environ Res Public Health. 2021;18(10). 10.3390/ijerph18105360 PMC8157390 34069883

[49] Bilici Salman R, Gülbahar Ateş S, Satiş H, Tufan A, Akdemir Ü, Yapar D (2021). Diagnostic role of 18F-Fluorodeoxyglucose Positron Emission Tomography for the evaluation of patients with inflammation of unknown origin. J Clin Rheumatology: Practical Rep Rheumatic Musculoskelet Dis.

[50] Okuyucu K, Alagoz E, Demirbas S, Ince S, Karakas A, Karacalioglu O (2018). Evaluation of predictor variables of diagnostic [18F] FDG-PET/CT in fever of unknown origin. Q J Nuclear Med Mol Imaging: Official Publication Italian Association Nuclear Med (AIMN) [and] Int Association Radiopharmacology (IAR) [and] Sect So.

[51] Knappe LM, Verburg FA, Giovanella L, Luster M, Librizzi D (2023). Diagnostic value of FDG-PET/CT in the diagnostic work-up of inflammation of unknown origin. Nuklearmedizin.

[52] Weitzer F, Nazerani Hooshmand T, Pernthaler B, Sorantin E, Aigner RM (2022). Diagnostic value of F-18 FDG PET/CT in fever or inflammation of unknown origin in a large single-center retrospective study. Sci Rep.

[53] Gafter-Gvili A, Raibman S, Grossman A, Avni T, Paul M, Leibovici L et al. [18F]FDG-PET/CT for the diagnosis of patients with fever of unknown origin. QJM: Monthly J Association Physicians. 2014. 10.1093/qjmed/hcu193 25208896

[54] Tsuzuki S, Watanabe A, Iwata M, Toyama H, Terasawa T (2021). Predictors of diagnostic contributions and spontaneous remission of symptoms Associated with Positron Emission Tomography with Fluorine-18-Fluorodeoxy glucose combined with computed tomography in Classic Fever or inflammation of unknown origin: a retrospective study. J Korean Med Sci.

[55] Balink H, Collins J, Bruyn GA, Gemmel F (2009). F-18 FDG PET/CT in the diagnosis of fever of unknown origin. Clin Nucl Med.

[56] Sathekge M, Maes A, Kgomo M, Van de Wiele C (2010). Fluorodeoxyglucose uptake by lymph nodes of HIV patients is inversely related to CD4 cell count. Nucl Med Commun.

[57] Iyengar S, Chin B, Margolick JB, Sabundayo BP, Schwartz DH (2003). Anatomical loci of HIV-associated immune activation and association with viraemia. Lancet.

[58] Lucignani G (2013). Clinical and translational imaging: reviews in nuclear medicine and molecular imaging. Clin Translational Imaging.

[59] Wright WF, Mulders-Manders CM, Auwaerter PG, Bleeker-Rovers CP (2022). Fever of unknown origin (FUO) - a call for New Research standards and updated clinical management. Am J Med.

[60] Haidar G, Singh N (2022). Fever of unknown origin. N Engl J Med.

[61] Boellaard R, Delgado-Bolton R, Oyen WJ, Giammarile F, Tatsch K, Eschner W (2015). FDG PET/CT: EANM procedure guidelines for tumour imaging: version 2.0. Eur J Nucl Med Mol Imaging.

[62] Jamar F, Buscombe J, Chiti A, Christian PE, Delbeke D, Donohoe KJ (2013). EANM/SNMMI guideline for 18F-FDG use in inflammation and infection. J Nuclear Medicine: Official Publication Soc Nuclear Med.

[63] Slart RHJA, Glaudemans AWJM, Gheysens O, Lubberink M, Kero T, Dweck MR (2021). Procedural recommendations of cardiac PET/CT imaging: standardization in inflammatory-, infective-, infiltrative-, and innervation (4Is)-related cardiovascular diseases: a joint collaboration of the EACVI and the EANM. Eur J Nucl Med Mol Imaging.

[64] Hess S, Scholtens AM, Gormsen LC (2020). Patient Preparation and patient-related challenges with FDG-PET/CT in Infectious and Inflammatory Disease. PET Clin.

[65] Pijl JP, Glaudemans A, Gheysens O, Slart R, Kwee TC (2023). Importance of blood glucose management before (18)F-FDG PET/CT in 322 patients with bacteremia of unknown origin. Journal of nuclear medicine: official publication. Soc Nuclear Med.

[66] Nielsen AW, Hansen IT, Nielsen BD, Kjær SG, Blegvad-Nissen J, Rewers K et al. The effect of prednisolone and a short-term prednisolone discontinuation for the diagnostic accuracy of FDG-PET/CT in polymyalgia rheumatica-a prospective study of 101 patients. Eur J Nucl Med Mol Imaging. 2024. 10.1007/s00259-024-06697-8 PMC11224098 38563881

[67] Nielsen BD, Gormsen LC, Hansen IT, Keller KK, Therkildsen P, Hauge EM (2018). Three days of high-dose glucocorticoid treatment attenuates large-vessel 18F-FDG uptake in large-vessel giant cell arteritis but with a limited impact on diagnostic accuracy. Eur J Nucl Med Mol Imaging.

[68] Taimen K, Salomäki SP, Hohenthal U, Mali M, Kajander S, Seppänen M et al. The clinical impact of using (18)F-FDG-PET/CT in the diagnosis of suspected vasculitis: the effect of dose and timing of glucocorticoid treatment. Contrast Media Mol I. 2019;2019:9157637. 10.1155/2019/9157637 PMC6735179 31531005

[69] Pijl JP, Glaudemans A, Slart R, Yakar D, Wouthuyzen-Bakker M, Kwee TC (2019). FDG-PET/CT for detecting an infection focus in patients with bloodstream infection: factors affecting diagnostic yield. Clin Nucl Med.

[70] Kagna O, Kurash M, Ghanem-Zoubi N, Keidar Z, Israel O (2017). Does Antibiotic Treatment affect the diagnostic accuracy of (18)F-FDG PET/CT studies in patients with suspected infectious processes? Journal of nuclear medicine: official publication. Soc Nuclear Med.

[71] Franzius C, Juergens KU, Schober O (2006). Is PET/CT necessary in paediatric oncology? For. Eur J Nucl Med Mol Imaging.

[72] Georga S, Exadaktylou P, Petrou I, Katsampoukas D, Mpalaris V, Moralidis EI et al. Diagnostic value of (18)F-FDG-PET/CT in patients with FUO. J Clin Med. 2020;9(7). 10.3390/jcm9072112 PMC7408628 32635566

[73] Tokmak H, Ergonul O, Demirkol O, Cetiner M, Ferhanoglu B (2014). Diagnostic contribution of (18)F-FDG-PET/CT in fever of unknown origin. Int J Infect Diseases: IJID: Official Publication Int Soc Infect Dis.

[74] Zhu W, Cao W, Zheng X, Li X, Li Y, Chen B (2020). The diagnostic value of (18)F-FDG PET/CT in identifying the causes of fever of unknown origin. Clin Med.

[75] Eynath Y, Halperin E, Buchrits S, Gafter-Gvili A, Bernstine H, Catalano O et al. Predictors for spontaneous resolution of classical FUO in patients undergoing PET-CT. Intern Emerg Med. 2022. 10.1007/s11739-022-03171-x 36512183

[76] Chen J, Xing M, Xu D, Xie N, Zhang W, Ruan Q (2022). Diagnostic models for fever of unknown origin based on (18)F-FDG PET/CT: a prospective study in China. EJNMMI Res.

[77] Ferda J, Ferdova E, Zahlava J, Matejovic M, Kreuzberg B (2010). Fever of unknown origin: a value of (18)F-FDG-PET/CT with integrated full diagnostic isotropic CT imaging. Eur J Radiol.

[78] Singh N, Kumar R, Malhotra A, Bhalla AS, Kumar U, Sood R (2015). Diagnostic utility of fluorodeoxyglucose positron emission tomography/computed tomography in pyrexia of unknown origin. Indian J Nuclear Medicine: IJNM: Official J Soc Nuclear Med India.

[79] Pelosi E, Skanjeti A, Penna D, Arena V (2011). Role of integrated PET/CT with [(1)(8)F]-FDG in the management of patients with fever of unknown origin: a single-centre experience. Radiol Med.

[80] Keidar Z, Gurman-Balbir A, Gaitini D, Israel O (2008). Fever of unknown origin: the role of 18F-FDG PET/CT. Journal of nuclear medicine: official publication. Soc Nuclear Med.

[81] Kubota K, Tanaka N, Miyata Y, Ohtsu H, Nakahara T, Sakamoto S (2021). Comparison of (18)F-FDG PET/CT and (67)Ga-SPECT for the diagnosis of fever of unknown origin: a multicenter prospective study in Japan. Ann Nucl Med.

[82] Ergul N, Halac M, Cermik TF, Ozaras R, Sager S, Onsel C (2011). The diagnostic role of FDG PET/CT in patients with fever of unknown origin. Mol Imaging Radionucl Therapy.

[83] Takeuchi M, Dahabreh IJ, Nihashi T, Iwata M, Varghese GM, Terasawa T (2016). Nuclear Imaging for Classic Fever of unknown origin: Meta-Analysis. Journal of nuclear medicine: official publication. Soc Nuclear Med.

[84] Huang CK, Huang JY, Ruan SY, Chien KL (2020). Diagnostic performance of FDG PET/CT in critically ill patients with suspected infection: a systematic review and meta-analysis. J Formos Med Assoc.

[85] Li Q, Tian R, Wang H, Li L, Wu T, Ren Y (2022). Quantifying the contribution of (18)F-FDG PET to the diagnostic assessment of pediatric patients with fever of unknown origin: a systematic review and meta-analysis. Pediatr Radiol.

[86] Becerra Nakayo EM, Garcia Vicente AM, Soriano Castrejon AM, Mendoza Narvaez JA, Talavera Rubio MP, Poblete Garcia VM (2012). [Analysis of cost-effectiveness in the diagnosis of fever of unknown origin and the role of (18)F-FDG PET-CT: a proposal of diagnostic algorithm]. Revista Esp De Med Nuclear e Imagen Mol.

[87] Buch-Olsen KM, Andersen RV, Hess S, Braad PE, Schifter S (2014). 18F-FDG-PET/CT in fever of unknown origin: clinical value. Nucl Med Commun.

[88] Chen JC, Wang Q, Li Y, Zhao YY, Gao P, Qiu LH (2022). Current situation and cost-effectiveness of (18)F-FDG PET/CT for the diagnosis of fever of unknown origin and inflammation of unknown origin: a single-center, large-sample study from China. Eur J Radiol.

[89] Gafter-Gvili A, Raibman S, Grossman A, Avni T, Paul M, Leibovici L (2015). [18F]FDG-PET/CT for the diagnosis of patients with fever of unknown origin. QJM: Monthly J Association Physicians.

[90] Hung BT, Wang PW, Su YJ, Huang WC, Chang YH, Huang SH (2017). The efficacy of (18)F-FDG PET/CT and (67)Ga SPECT/CT in diagnosing fever of unknown origin. Int J Infect Diseases: IJID: Official Publication Int Soc Infect Dis.

[91] Jaruskova M, Belohlavek O (2006). Role of FDG-PET and PET/CT in the diagnosis of prolonged febrile states. Eur J Nucl Med Mol Imaging.

[92] Kei PL, Kok TY, Padhy AK, Ng DC, Goh AS (2010). [18F] FDG PET/CT in patients with fever of unknown origin: a local experience. Nucl Med Commun.

[93] Manohar K, Mittal BR, Jain S, Sharma A, Kalra N, Bhattacharya A (2013). F-18 FDG-PET/CT in evaluation of patients with fever of unknown origin. Japanese J Radiol.

[94] Pedersen TI, Roed C, Knudsen LS, Loft A, Skinhoj P, Nielsen SD (2012). Fever of unknown origin: a retrospective study of 52 cases with evaluation of the diagnostic utility of FDG-PET/CT. Scand J Infect Dis.

[95] Sheng JF, Sheng ZK, Shen XM, Bi S, Li JJ, Sheng GP (2011). Diagnostic value of fluorine-18 fluorodeoxyglucose positron emission tomography/computed tomography in patients with fever of unknown origin. Eur J Intern Med.

[96] Tokmak H, Ergonul O, Demirkol O, Cetiner M, Ferhanoglu B. Diagnostic contribution of F-FDG-PET/CT in fever of unknown origin. International journal of infectious diseases: IJID: official publication of the International Society for Infectious Diseases. 2013. 10.1016/j.ijid.2013.10.009 24295559

[97] Nygaard U, Larsen LV, Vissing NH, von Linstow ML, Myrup C, Berthelsen AK (2022). Unexplained fever in children-benefits and challenges of FDG-PET/CT. Acta Paediatr.

[98] Chang L, Cheng MF, Jou ST, Ko CL, Huang JY, Tzen KY (2016). Search of unknown fever focus using PET in critically Ill Children with Complicated Underlying diseases. Pediatr Crit Care Med.

[99] Yang J, Zhuang H (2015). The role of 18F-FDG PET/CT in the evaluation of pediatric transplant patients. Hell J Nucl Med.

[100] Blokhuis GJ, Bleeker-Rovers CP, Diender MG, Oyen WJ, Draaisma JM, de Geus-Oei LF (2014). Diagnostic value of FDG-PET/(CT) in children with fever of unknown origin and unexplained fever during immune suppression. Eur J Nucl Med Mol Imaging.

[101] Simons KS, Pickkers P, Bleeker-Rovers CP, Oyen WJ, van der Hoeven JG (2010). F-18-fluorodeoxyglucose positron emission tomography combined with CT in critically ill patients with suspected infection. Intensive Care Med.

[102] Tek Chand K, Chennu KK, Amancharla Yadagiri L, Manthri Gupta R, Rapur R, Vishnubotla SK. Utility of 18 F-FDG PET/CT scan to diagnose the etiology of fever of unknown origin in patients on dialysis. Hemodialysis international International Symposium on Home Hemodialysis. 2017;21(2):224 – 31. 10.1111/hdi.12471 27616744

[103] Castaigne C, Tondeur M, de Wit S, Hildebrand M, Clumeck N, Dusart M (2009). Clinical value of FDG-PET/CT for the diagnosis of human immunodeficiency virus-associated fever of unknown origin: a retrospective study. Nucl Med Commun.

[104] Knockaert DC, Dujardin KS, Bobbaers HJ (1996). Long-term follow-up of patients with undiagnosed fever of unknown origin. Arch Intern Med.

[105] Tan Y, Liu X, Shi X (2019). Clinical features and outcomes of patients with fever of unknown origin: a retrospective study. BMC Infect Dis.

[106] Mulders-Manders CM, Engwerda C, Simon A, van der Meer JWM, Bleeker-Rovers CP (2018). Long-term prognosis, treatment, and outcome of patients with fever of unknown origin in whom no diagnosis was made despite extensive investigation: a questionnaire based study. Medicine.

[107] Statler VA, Marshall GS (2016). Characteristics of patients referred to a Pediatric Infectious diseases Clinic with unexplained fever. J Pediatr Infect Dis Soc.

[108] Berrevoets MAH, Kouijzer IJE, Aarntzen E, Janssen MJR, De Geus-Oei LF, Wertheim HFL (2017). 18)F-FDG PET/CT optimizes treatment in Staphylococcus Aureus Bacteremia and is Associated with reduced mortality. Journal of nuclear medicine: official publication. Soc Nuclear Med.

[109] Vos FJ, Bleeker-Rovers CP, Kullberg BJ, Adang EM, Oyen WJ (2011). Cost-effectiveness of routine (18)F-FDG PET/CT in high-risk patients with gram-positive bacteremia. J Nuclear Medicine: Official Publication Soc Nuclear Med.

[110] Diemberger I, Bonfiglioli R, Martignani C, Graziosi M, Biffi M, Lorenzetti S (2019). Contribution of PET imaging to mortality risk stratification in candidates to lead extraction for pacemaker or defibrillator infection: a prospective single center study. Eur J Nucl Med Mol Imaging.

[111] Balink H, Tan SS, Veeger NJ, Holleman F, van Eck-Smit BL, Bennink RJ (2015). 1)(8)F-FDG PET/CT in inflammation of unknown origin: a cost-effectiveness pilot-study. Eur J Nucl Med Mol Imaging.

