# Supplementary material for: EANM consensus document on the use of [18F]FDG PET/CT in fever and inflammation of unknown origin
Source: Eur J Nucl Med Mol Imaging. 2024 Apr 27;51(9):2597–613. doi: 10.1007/s00259-024-06732-8 (PMC11224117; doi:10.1007/s00259-024-06732-8)
Supplement: Supplementary file 1 — Supplementary Material 1 [file 259_2024_6732_MOESM1_ESM.docx]

**SUPPLEMENTAL FILE 1. PubMed search string.**

(("fever of unknown origin"[MeSH Terms] OR ("fever"[All Fields] AND "unknown"[All Fields] AND "origin"[All Fields]) OR "fever of unknown origin"[All Fields] OR ("fever of unknown origin"[MeSH Terms] OR ("fever"[All Fields] AND "unknown"[All Fields] AND "origin"[All Fields]) OR "fever of unknown origin"[All Fields] OR ("pyrexia"[All Fields] AND "unknown"[All Fields] AND "origin"[All Fields]) OR "pyrexia of unknown origin"[All Fields]) OR (("inflammation"[MeSH Terms] OR "inflammation"[All Fields] OR "inflammations"[All Fields] OR "inflammation s"[All Fields]) AND ("unknown"[All Fields] OR "unknowns"[All Fields]) AND ("origin"[All Fields] OR "originate"[All Fields] OR "originated"[All Fields] OR "originates"[All Fields] OR "originating"[All Fields] OR "origination"[All Fields] OR "originations"[All Fields] OR "origins"[All Fields])) OR "FUO"[All Fields] OR "PUO"[All Fields] OR "IUO"[All Fields] OR "fever of unknown origin"[MeSH Terms] OR "fever of unknown origin"[MeSH Terms] OR "fever of unknown origin/diagnosis"[MeSH Terms] OR "fever of unknown origin/diagnostic imaging"[MeSH Terms] OR ("fever"[MeSH Terms] OR "fever"[All Fields] OR "fevers"[All Fields] OR ("fever"[MeSH Terms] OR "fever"[All Fields] OR "pyrexia"[All Fields] OR "pyrexias"[All Fields]) OR ("inflammation"[MeSH Terms] OR "inflammation"[All Fields] OR "inflammations"[All Fields] OR "inflammation s"[All Fields]) OR "fever"[MeSH Terms] OR "inflammation"[MeSH Terms] OR "fever"[MeSH Terms])) AND ("fdg"[Journal] OR "fdg"[All Fields] OR ("fluorodeoxyglucose f18"[MeSH Terms] OR ("fluorodeoxyglucose"[All Fields] AND "f18"[All Fields]) OR "fluorodeoxyglucose f18"[All Fields] OR "fluorodeoxyglucose"[All Fields]) OR "fluorodeoxyglucose f18"[MeSH Terms] OR ("positron emission tomography computed tomography"[MeSH Terms] OR ("positron"[All Fields] AND "emission"[All Fields] AND "tomography"[All Fields] AND "computed"[All Fields]) OR "positron emission tomography computed tomography"[All Fields] OR ("pet"[All Fields] AND "ct"[All Fields]) OR "pet ct"[All Fields] OR ("positron emission tomography computed tomography"[MeSH Terms] OR ("positron"[All Fields] AND "emission"[All Fields] AND "tomography"[All Fields] AND "computed"[All Fields]) OR "positron emission tomography computed tomography"[All Fields] OR ("pet"[All Fields] AND "ct"[All Fields]) OR "pet ct"[All Fields]) OR "positron emission tomography computed tomography"[All Fields] OR "positron emission tomography computed tomography"[MeSH Terms] OR "positron emission tomography computed tomography"[MeSH Terms] OR "positron emission tomography computed tomography"[MeSH Terms]))) AND ((humans[Filter]) AND (english[Filter]) AND (2001:2024[pdat]))
