# Supplementary figures and images for: EANM consensus document on the use of [18F]FDG PET/CT in fever and inflammation of unknown origin
Source: Eur J Nucl Med Mol Imaging. 2024 Apr 27;51(9):2597–613. doi: 10.1007/s00259-024-06732-8 (PMC11224117; doi:10.1007/s00259-024-06732-8)

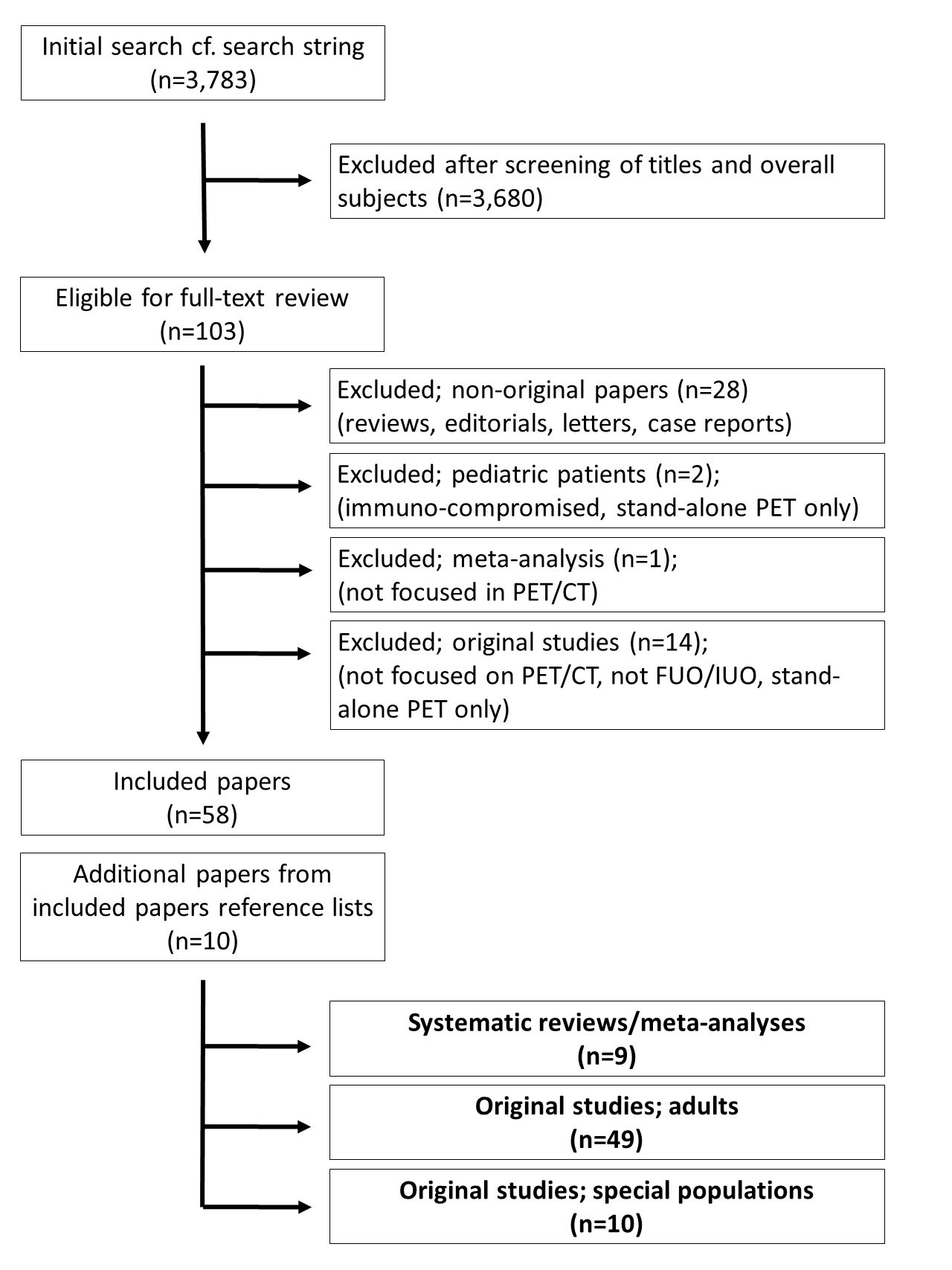

Supplement: Supplementary file 2 — Supplementary Material 2 [file 259_2024_6732_MOESM2_ESM.png]
