# Supplementary material for: EANM consensus document on the use of [18F]FDG PET/CT in fever and inflammation of unknown origin
Source: Eur J Nucl Med Mol Imaging. 2024 Apr 27;51(9):2597–613. doi: 10.1007/s00259-024-06732-8 (PMC11224117; doi:10.1007/s00259-024-06732-8)
Supplement: Supplementary file 3 — Supplementary Material 3 [file 259_2024_6732_MOESM3_ESM.docx]

**SUPPLEMENTAL FILE 3.**

**List of included papers on the role of [^18^F]FDG PET/CT in patients with fever of unknown origin; listed in reverse chronological order of publication.**

**Systematic reviews and meta-analyses.**

*Adults*

Kan Y, Wang W, Liu J, Yang J, Wang Z. Contribution of 18F-FDG PET/CT in a case-mix of fever of unknown origin and inflammation of unknown origin: a meta-analysis. Acta Radiol. 2019 Jun;60(6):716-725.

PMID: 30205705.

Takeuchi M, Nihashi T, Gafter-Gvili A, García-Gómez FJ, Andres E, Blockmans D, Iwata M, Terasawa T. Association of 18F-FDG PET or PET/CT results with spontaneous remission in classic fever of unknown origin: A systematic review and meta-analysis. Medicine (Baltimore). 2018 Oct;97(43):e12909.

PMID: 30412093.

Bharucha T, Rutherford A, Skeoch S, Alavi A, Brown M, Galloway J; FDG-PET/CT in fever of unknown origin working group. Diagnostic yield of FDG-PET/CT in fever of unknown origin: a systematic review, meta-analysis, and Delphi exercise. Clin Radiol. 2017 Sep;72(9):764-771. PMID: 28600002.

Takeuchi M, Dahabreh IJ, Nihashi T, Iwata M, Varghese GM, Terasawa T. Nuclear Imaging for Classic Fever of Unknown Origin: Meta-Analysis. J Nucl Med. 2016 Dec;57(12):1913-1919. PMID: 27339873.

Besson FL, Chaumet-Riffaud P, Playe M, Noel N, Lambotte O, Goujard C, Prigent A, Durand E. Contribution of (18)F-FDG PET in the diagnostic assessment of fever of unknown origin (FUO): a stratification-based meta-analysis. Eur J Nucl Med Mol Imaging. 2016 Sep;43(10):1887-95. PMID: 27037917.

Hao R, Yuan L, Kan Y, Li C, Yang J. Diagnostic performance of 18F-FDG PET/CT in patients with fever of unknown origin: a meta-analysis. Nucl Med Commun. 2013 Jul;34(7):682-8. PMID: 23636293.

Dong MJ, Zhao K, Liu ZF, Wang GL, Yang SY, Zhou GJ. A meta-analysis of the value of fluorodeoxyglucose-PET/PET-CT in the evaluation of fever of unknown origin. Eur J Radiol. 2011 Dec;80(3):834-44. PMID: 21131151.

*Special populations*

Li Q, Tian R, Wang H, Li L, Wu T, Ren Y, Su M, Zou K, Sun X. Quantifying the contribution of <sup>18</sup>F-FDG PET to the diagnostic assessment of pediatric patients with fever of unknown origin: a systematic review and meta-analysis. Pediatr Radiol. 2022 Jul;52(8):1500-1511. PMID: 35348809.

Huang CK, Huang JY, Ruan SY, Chien KL. Diagnostic performance of FDG PET/CT in critically ill patients with suspected infection: A systematic review and meta-analysis. J Formos Med Assoc. 2020 May;119(5):941-949. PMID: 31604648.

**Original papers.**

Akyüz Dağlı P, Güven SC, Coşkun N, Karakaş Ö, Armağan B, Orhan K, Doğan İ, Maraş Y, Türkölmez Ş, Erten Ş. Rheumatology experience with FDG PET / CT in inflammation of unknown origin: a single - centre report for determining factors associated with diagnostic precision. Clin Rheumatol. 2023 Oct;42(10):2861-2872. PMID: 37338744.

Betrains A, Boeckxstaens L, Moreel L, Wright WF, Blockmans D, Van Laere K, Vanderschueren S. Higher diagnostic yield of 18F-FDG PET in inflammation of unknown origin compared to fever of unknown origin. Eur J Intern Med. 2023 Apr;110:71-76. PMID: 36739216.

Knappe LM, Verburg FA, Giovanella L, Luster M, Librizzi D. Diagnostic value of FDG-PET/CT in the diagnostic work-up of inflammation of unknown origin. Nuklearmedizin. 2023 Feb;62(1):27-33. PMID: 36623829.

Eynath Y, Halperin E, Buchrits S, Gafter-Gvili A, Bernstine H, Catalano O, Avni T. Predictors for spontaneous resolution of classical FUO in patients undergoing PET-CT. Intern Emerg Med. 2023 Mar;18(2):367-374. PMID: 36512183.

Holubar J, Broner J, Arnaud E, Hallé O, Mura T, Chambert B, Sotto A, Roubille C, Gaujoux-Viala C, Goulabchand R. Diagnostic performance of <sup>18</sup>F-FDG-PET/CT in inflammation of unknown origin: A clinical series of 317 patients. J Intern Med. 2022 Jun;291(6):856-863. PMID: 35018669.

Chen JC, Wang Q, Li Y, Zhao YY, Gao P, Qiu LH, Hao KJ, Li HB, Yue MG, Zhou YS, Zhu JH, Gao Y, Gao ZC. Current situation and cost-effectiveness of <sup>18</sup>F-FDG PET/CT for the diagnosis of fever of unknown origin and inflammation of unknown origin: A single-center, large-sample study from China. Eur J Radiol. 2022 Mar;148:110184. PMID: 35121332.

Chen Z, Li Y, Wang Q, Weng S, Zhou Y, Zhu J. Fluorine-18 labeled fluorodeoxyglucose positron emission tomography/computed tomography used in diagnosing connective tissue diseases in fever of unknown origin/inflammatory of unknown origin patients. Clin Rheumatol. 2022 Mar;41(3):839-846. PMID: 34674082.

Weitzer F, Nazerani Hooshmand T, Pernthaler B, Sorantin E, Aigner RM. Diagnostic value of F-18 FDG PET/CT in fever or inflammation of unknown origin in a large single-center retrospective study. Sci Rep. 2022 Feb 3;12(1):1883. PMID: 35115619.

Ly KH, Costedoat-Chalumeau N, Liozon E, Dumonteil S, Ducroix JP, Sailler L, Lidove O, Bienvenu B, Decaux O, Hatron PY, Smail A, Astudillo L, Morel N, Boutemy J, Perlat A, Denes E, Lambert M, Papo T, Cypierre A, Vidal E, Preux PM, Monteil J, Fauchais AL. Diagnostic Value of 18F-FDG PET/CT vs. Chest-Abdomen-Pelvis CT Scan in Management of Patients with Fever of Unknown Origin, Inflammation of Unknown Origin or Episodic Fever of Unknown Origin: A Comparative Multicentre Prospective Study. J Clin Med. 2022 Jan 13;11(2):386. PMID: 35054081.

Buchrits S, Gafter-Gvili A, Eynath Y, Bernstine H, Guz D, Avni T. The yield of F<sup>18</sup> FDG PET-CT for the investigation of fever of unknown origin, compared with diagnostic CT. Eur J Intern Med. 2021 Nov;93:50-56. PMID: 34420847.

Bilici Salman R, Gülbahar Ateş S, Satiş H, Tufan A, Akdemir ÜÖ, Yapar D, Ataş N, Güler AA, Karadeniz H, Babaoglu H, Aydos U, Göker B, Haznedaroğlu Ş, Atay LÖ, Öztürk MA. Diagnostic Role of 18F-Fluorodeoxyglucose Positron Emission Tomography for the Evaluation of Patients With Inflammation of Unknown Origin. J Clin Rheumatol. 2021 Sep 1;27(6):219-225. PMID: 32195847.

Tsuzuki S, Watanabe A, Iwata M, Toyama H, Terasawa T. Predictors of Diagnostic Contributions and Spontaneous Remission of Symptoms Associated with Positron Emission Tomography with Fluorine-18-Fluorodeoxy Glucose Combined with Computed Tomography in Classic Fever or Inflammation of Unknown Origin: a Retrospective Study. J Korean Med Sci. 2021 Jun 7;36(22):e150. PMID: 34100562.

Mahajna H, Vaknin K, Ben Shimol J, Watad A, Abu-Much A, Mahroum N, Shovman O, Shoenfeld Y, Amital H, Davidson T. The Utility of 18FDG-PET/CT in Diagnosing Fever of Unknown Origin: The Experience of a Large Tertiary Medical Center. Int J Environ Res Public Health. 2021 May 18;18(10):5360. PMID: 34069883.

Kubota K, Tanaka N, Miyata Y, Ohtsu H, Nakahara T, Sakamoto S, Kudo T, Nishiyama Y, Tateishi U, Murakami K, Nakamoto Y, Taki Y, Kaneta T, Kawabe J, Nagamachi S, Kawano T, Hatazawa J, Mizutani Y, Baba S, Kirii K, Yokoyama K, Okamura T, Kameyama M, Minamimoto R, Kunimatsu J, Kato O, Yamashita H, Kaneko H,

Kutsuna S, Ohmagari N, Hagiwara A, Kikuchi Y, Kobayakawa M. Comparison of <sup>18</sup>F-FDG PET/CT and <sup>67</sup>Ga-SPECT for the diagnosis of fever of unknown origin: a multicenter prospective study in Japan. Ann Nucl Med. 2021 Jan;35(1):31-46. PMID: 33037581.

Zhu W, Cao W, Zheng X, Li X, Li Y, Chen B, Zhang J. The diagnostic value of <sup>18</sup>F-FDG PET/CT in identifying the causes of fever of unknown origin. Clin Med (Lond). 2020 Sep;20(5):449-453. PMID: 32934036.

Georga S, Exadaktylou P, Petrou I, Katsampoukas D, Mpalaris V, Moralidis EI, Arvaniti K, Papastergiou C, Arsos G. Diagnostic Value of ^18^F-FDG-PET/CT in Patients with FUO. J Clin Med. 2020 Jul 4;9(7):2112. PMID: 32635566.

Wang WX, Cheng ZT, Zhu JL, Xing MY, Zheng CF, Wang SJ, Xie NN, XianYu ZQ, Song JX. Combined clinical parameters improve the diagnostic efficacy of <sup>18</sup>F-FDG PET/CT in patients with fever of unknown origin (FUO) and inflammation of unknown origin (IUO): A prospective study in China. Int J Infect

Dis. 2020 Apr;93:77-83. PMID: 31982625.

Wang Q, Li YM, Li Y, Hua FC, Wang QS, Zhang XL, Cheng C, Wu H, Yao ZM, Zhang WF, Hou QY, Miao WB, Wang XM. <sup>18</sup>F-FDGPET/CT in fever of unknown origin and inflammation of unknown origin: a Chinese multi-center study. Eur J Nucl Med Mol Imaging. 2019 Jan;46(1):159-165. PMID: 30099578.

Okuyucu K, Alagoz E, Demirbas S, Ince S, Karakas A, Karacalioglu O, Gunalp B, Arslan N. Evaluation of predictor variables of diagnostic [18F] FDG-PET/CT in fever of unknown origin. Q J Nucl Med Mol Imaging. 2018 Sep;62(3):313-320. PMID: 26554525.

García-Vicente AM, Tello-Galán MJ, Amo-Salas M, Ros-Izquierdo J, Jiménez-Londoño GA, La Rosa Salas B, Prado-Serrano Pradas G, Pena-Pardo FJ, Soriano-Castrejón Á. Do clinical and laboratory variables have any impact on the diagnostic performance of 18F-FDG PET/CT in patients with fever of unknown origin? Ann Nucl Med. 2018 Feb;32(2):123-131. PMID: 29264739.

Schönau V, Vogel K, Englbrecht M, Wacker J, Schmidt D, Manger B, Kuwert T, Schett G. The value of <sup>18</sup>F-FDG-PET/CT in identifying the cause of fever of unknown origin (FUO) and inflammation of unknown origin (IUO): data from a prospective study. Ann Rheum Dis. 2018 Jan;77(1):70-77. PMID: 28928271.

Hung BT, Wang PW, Su YJ, Huang WC, Chang YH, Huang SH, Chang CC. The efficacy of <sup>18</sup>F-FDG PET/CT and <sup>67</sup>Ga SPECT/CT in diagnosing fever of unknown origin. Int J Infect Dis. 2017 Sep;62:10-17. PMID: 28652213.

Bouter C, Braune I, Meller B, Sahlmann C, Ritter C, Meller J. <sup>18</sup>F-FDG-PET/CT in unexplained elevated inflammatory markers. Joining entities. Nuklearmedizin. 2016 Dec 6;55(6):242-249. doi:

10.3413/Nukmed-0798-16-02. Epub 2016 Sep 12. PMID: 27617327.

Pereira AM, Husmann L, Sah BR, Battegay E, Franzen D. Determinants of diagnostic performance of 18F-FDG PET/CT in patients with fever of unknown origin. Nucl Med Commun. 2016 Jan;37(1):57-65. PMID: 26426966.

Balink H, Veeger NJ, Bennink RJ, Slart RH, Holleman F, van Eck-Smit BL, Verberne HJ. The predictive value of C-reactive protein and erythrocyte sedimentation rate for 18F-FDG PET/CT outcome in patients with fever and inflammation of unknown origin. Nucl Med Commun. 2015 Jun;36(6):604-9. PMID: 25738560.

Gafter-Gvili A, Raibman S, Grossman A, Avni T, Paul M, Leibovici L, Tadmor B, Groshar D, Bernstine H. [18F]FDG-PET/CT for the diagnosis of patients with fever of unknown origin. QJM. 2015 Apr;108(4):289-98. PMID: 25208896.

Buch-Olsen KM, Andersen RV, Hess S, Braad PE, Schifter S. 18F-FDG-PET/CT in fever of unknown origin: clinical value. Nucl Med Commun. 2014 Sep;35(9):955-60. PMID: 24801131.

Balink H, Bennink RJ, Veeger NJ, van Eck-Smit BL, Verberne HJ. Diagnostic utility of (18)F-FDG PET/CT in inflammation of unknown origin. Clin Nucl Med. 2014 May;39(5):419-25. PMID: 24662652.

Tokmak H, Ergonul O, Demirkol O, Cetiner M, Ferhanoglu B. Diagnostic contribution of (18)F-FDG-PET/CT in fever of unknown origin. Int J Infect Dis. 2014 Feb;19:53-8. PMID: 24295559.

Manohar K, Mittal BR, Jain S, Sharma A, Kalra N, Bhattacharya A, Varma S. F-18 FDG-PET/CT in evaluation of patients with fever of unknown origin. Jpn J Radiol. 2013 May;31(5):320-7. PMID: 23456545.

Crouzet J, Boudousq V, Lechiche C, Pouget JP, Kotzki PO, Collombier L, Lavigne JP, Sotto A. Place of (18)F-FDG-PET with computed tomography in the diagnostic algorithm of patients with fever of unknown origin. Eur J Clin Microbiol Infect Dis. 2012 Aug;31(8):1727-33. PMID: 22735898.

Kim YJ, Kim SI, Hong KW, Kang MW. Diagnostic value of 18F-FDG PET/CT in patients with fever of unknown origin. Intern Med J. 2012 Jul;42(7):834-7. PMID: 22805689.

Pedersen TI, Roed C, Knudsen LS, Loft A, Skinhoj P, Nielsen SD. Fever of unknown origin: a retrospective study of 52 cases with evaluation of the diagnostic utility of FDG-PET/CT. Scand J Infect Dis. 2012 Jan;44(1):18-23.

PMID: 21888563.

Pelosi E, Skanjeti A, Penna D, Arena V. Role of integrated PET/CT with [¹⁸F]-FDG in the management of patients with fever of unknown origin: a single-centre experience. Radiol Med. 2011 Aug;116(5):809-20. PMID: 21424320.

Kubota K, Nakamoto Y, Tamaki N, Kanegae K, Fukuda H, Kaneda T, Kitajima K, Tateishi U, Morooka M, Ito K, Minamimoto R, Murakami K. FDG-PET for the diagnosis of fever of unknown origin: a Japanese multi-center study. Ann Nucl Med. 2011 Jun;25(5):355-64. PMID: 21344168.

Sheng JF, Sheng ZK, Shen XM, Bi S, Li JJ, Sheng GP, Yu HY, Huang HJ, Liu J, Xiang DR, Dong MJ, Zhao K, Li LJ. Diagnostic value of fluorine-18 fluorodeoxyglucose positron emission tomography/computed tomography in patients with fever of unknown origin. Eur J Intern Med. 2011 Feb;22(1):112-6. PMID: 21238906.

Kei PL, Kok TY, Padhy AK, Ng DC, Goh AS. [18F] FDG PET/CT in patients with fever of unknown origin: a local experience. Nucl Med Commun. 2010 Sep;31(9):788-92. PMID: 20634769.

Ferda J, Ferdová E, Záhlava J, Matejovic M, Kreuzberg B. Fever of unknown origin: a value of (18)F-FDG-PET/CT with integrated full diagnostic isotropic CT imaging. Eur J Radiol. 2010 Mar;73(3):518-25. PMID: 19201122.

Federici L, Blondet C, Imperiale A, Sibilia J, Pasquali JL, Pflumio F, Goichot B, Blaison G, Weber JC, Christmann D, Constantinesco A, Andrès E. Value of (18)F-FDG-PET/CT in patients with fever of unknown origin and unexplained prolonged inflammatory syndrome: a single centre analysis experience. Int J Clin Pract. 2010 Jan;64(1):55-60. PMID: 18479364.

Balink H, Collins J, Bruyn GA, Gemmel F. F-18 FDG PET/CT in the diagnosis of fever of unknown origin. Clin Nucl Med. 2009 Dec;34(12):862-8. PMID: 20139818.

Keidar Z, Gurman-Balbir A, Gaitini D, Israel O. Fever of unknown origin: the role of 18F-FDG PET/CT. J Nucl Med. 2008 Dec;49(12):1980-5. PMID: 18997040.

Jaruskova M, Belohlavek O. Role of FDG-PET and PET/CT in the diagnosis of prolonged febrile states. Eur J Nucl Med Mol Imaging. 2006 Aug;33(8):913-8. PMID: 16572304.

**Original papers (special populations).**

*Children*

Nygaard U, Larsen LV, Vissing NH, von Linstow ML, Myrup C, Berthelsen AK, Poulsen A, Borgwardt L. Unexplained fever in children-Benefits and challenges of FDG-PET/CT. Acta Paediatr. 2022 Nov;111(11):2203-2209. PMID: 36210785.

Pijl JP, Kwee TC, Legger GE, Peters HJH, Armbrust W, Schölvinck EH, Glaudemans AWJM. Role of FDG-PET/CT in children with fever of unknown origin. Eur J Nucl Med Mol Imaging. 2020 Jun;47(6):1596-1604. PMID: 32030452.

Chang L, Cheng MF, Jou ST, Ko CL, Huang JY, Tzen KY, Yen RF. Search of Unknown Fever Focus Using PET in Critically Ill Children With Complicated Underlying Diseases. Pediatr Crit Care Med. 2016 Feb;17(2):e58-65. PMID: 26649939.

Blokhuis GJ, Bleeker-Rovers CP, Diender MG, Oyen WJ, Draaisma JM, de Geus-Oei LF. Diagnostic value of FDG-PET/(CT) in children with fever of unknown origin and unexplained fever during immune suppression. Eur J Nucl Med Mol Imaging. 2014 Oct;41(10):1916-23. PMID: 24869631.

Jasper N, Däbritz J, Frosch M, Loeffler M, Weckesser M, Foell D. Diagnostic value of [(18)F]-FDG PET/CT in children with fever of unknown origin or unexplained signs of inflammation. Eur J Nucl Med Mol Imaging. 2010 Jan;37(1):136-45. doi: 10.1007/s00259-009-1185-y. PMID: 19526234.

*Intensive Care Unit-patients*

Simons KS, Pickkers P, Bleeker-Rovers CP, Oyen WJ, van der Hoeven JG. F-18-fluorodeoxyglucose positron emission tomography combined with CT in critically ill patients with suspected infection. Intensive Care Med. 2010 Mar;36(3):504-11. PMID: 19847397.

*End-stage renal disease*

Lawal IO, Popoola GO, Lengana T, Ankrah AO, Ebenhan T, Sathekge MM. Diagnostic utility of <sup>18</sup>F-FDG PET/CT in fever of unknown origin among patients with end-stage renal disease treated with renal replacement therapy. Hell J Nucl Med. 2019 Jan-Apr;22(1):70-75. PMID: 30843013.

Tek Chand K, Chennu KK, Amancharla Yadagiri L, Manthri Gupta R, Rapur R, Vishnubotla SK. Utility of 18 F-FDG PET/CT scan to diagnose the etiology of fever of unknown origin in patients on dialysis. Hemodial Int. 2017 Apr;21(2):224-231. PMID: 27616744.

*Human Immunodeficiency Virus*

Martin C, Castaigne C, Tondeur M, Flamen P, De Wit S. Role and interpretation of fluorodeoxyglucose-positron emission tomography/computed tomography in HIV-infected patients with fever of unknown origin: a prospective study. HIV Med. 2013 Sep;14(8):455-62. PMID: 23517190.

Castaigne C, Tondeur M, de Wit S, Hildebrand M, Clumeck N, Dusart M. Clinical value of FDG-PET/CT for the diagnosis of human immunodeficiency virus-associated fever of unknown origin: a retrospective study. Nucl Med Commun. 2009 Jan;30(1):41-7. PMID: 19306513.
